# Structure-Function Characteristics of SARS-CoV-2 Proteases and Their Potential Inhibitors from Microbial Sources

**DOI:** 10.3390/microorganisms9122481

**Published:** 2021-11-30

**Authors:** Rafida Razali, Haslina Asis, Cahyo Budiman

**Affiliations:** Biotechnology Research Institute, Universiti Malaysia Sabah, Kota Kinabalu 88400, Sabah, Malaysia; rafidarazali@gmail.com (R.R.); haslinaasis01@gmail.com (H.A.)

**Keywords:** COVID-19, SARS-CoV-2 proteases, 3-chymotrypsin-like protease, papain-like protease, microbial, inhibitors

## Abstract

The COVID-19 pandemic, caused by Severe Acute Respiratory Syndrome Coronavirus 2 (SARS-CoV-2), is considered the greatest challenge to the global health community of the century as it continues to expand. This has prompted immediate urgency to discover promising drug targets for the treatment of COVID-19. The SARS-CoV-2 viral proteases, 3-chymotrypsin-like protease (3CLpro) and papain-like cysteine protease (PLpro), have become the promising target to study due to their essential functions in spreading the virus by RNA transcription, translation, protein synthesis, processing and modification, virus replication, and infection of the host. As such, understanding of the structure and function of these two proteases is unavoidable as platforms for the development of inhibitors targeting this protein which further arrest the infection and spread of the virus. While the abundance of reports on the screening of natural compounds such as SARS-CoV-2 proteases inhibitors are available, the microorganisms-based compounds (peptides and non-peptides) remain less studied. Indeed, microorganisms-based compounds are also one of the potent antiviral candidates against COVID-19. Microbes, especially bacteria and fungi, are other resources to produce new drugs as well as nucleosides, nucleotides, and nucleic acids. Thus, we have compiled various reported literature in detail on the structures, functions of the SARS-CoV-2 proteases, and potential inhibitors from microbial sources as assistance to other researchers working with COVID-19. The compounds are also compared to HIV protease inhibitors which suggested the microorganisms-based compounds are advantageous as SARS-CoV2 proteases inhibitors. The information should serve as a platform for further development of COVID-19 drug design strategies.

## 1. Introduction

In late December 2019, a new strain of coronavirus resulted in the outbreak of a pneumonia-like illness in Wuhan, China and has become a life-threatening concern worldwide in the present time [1,2]. Accordingly, WHO names the disease as the coronavirus disease 2019 (COVID-19) [1,2]. The new strain of this coronavirus has been named Severe Acute Respiratory Syndrome Coronavirus 2 (SARS-CoV-2) by the World Health Organization (WHO) [3] since the RNA genome is about 82% identical to that of the SARS coronavirus (SARS-CoV). A person with COVID-19 will usually suffer from fever, dry cough, tiredness, and shortness of breath under severe conditions; others may be just silent carriers of the virus [4]. Due to the high transmission efficiency of this new coronavirus, on 11 March 2020, WHO has officially declared the COVID-19 as a pandemic.

Coronaviruses (CoVs) are the largest group of viruses under the order Nidovirales, family Coronaviridae, and subfamily Orthocoronavirinae [5,6,7]. The Orthocoronavirinae are further classified into four genera: alphacoronavirus (α-CoV), betacoronavirus (β-CoV), gammacoronavirus (γ-CoV) and deltacoronavirus (δ-CoV), based on genetic and antigenic criteria [8]. All viruses in the Nidovirales order are spherical and enveloped with club-shaped spikes on the surface giving the appearance of a crown-like protrusion [8]. They are zoonotic viruses that infect various vertebrates (pets, bats, livestock, poultry, and human), and among human, CoVs are responsible for respiratory, gastrointestinal, and neurological problems [9,10]. They all contain very large RNA viruses’ genomes, with some having the largest identified RNA genomes [5]. The first decade of the 21st century has witnessed an increase in the number of CoVs that caused a major outbreak of fatal human pneumonia. In 2003, Severe Acute Respiratory Syndrome Coronavirus (SARS-CoV) broke out in five continents, and the Middle East Respiratory Syndrome Coronavirus (MERS-CoV) broke out in the Arabian Peninsula in 2011 with mortality rates of 9.6% and 35.5%, respectively [11,12,13]. To date, there is no specific therapeutic drug for the treatment of the human coronavirus, of which the outbreak poses a huge threat to humans.

**Figure 1:**
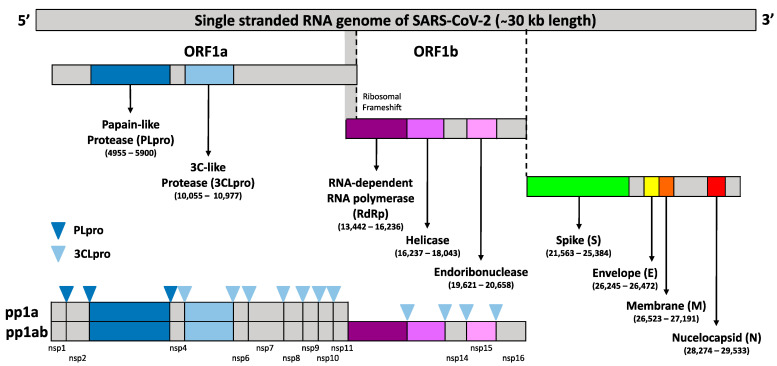
Schematic representation of single stranded SARS-CoV-2 genome structure with ∼30 kb nucleotides in length with a 5′cap structure and a 3′-poly(A) tail. The first ORF contains a frameshift in between a-1 of ORF1a and ORF1b which directly translated two polypeptides (pp1a and pp1ab). These polyproteins are processed by one or two papain-like proteases (PLpro) in which the dark blue upside-down triangle sign indicated the cleavage sites of 3CLpro, and by the 3C-like protease (3CLpro) in which the light blue upside-down triangle indicated the cleavage sites of 3CLpro, into the 16 nsps (nsp1–16).

The SARS-CoV-2 belongs to clade B of β-CoV genus and has a (+) sense ssRNA genome [14]. The (+) sense ssRNA genome of CoVs is ∼30,000 nucleotides in length with a 5′-cap structure and a 3′-poly(A) tail consisting of at least six open reading frames (ORFs) [15,16]. It contains several essential genes that encode the viral proteins necessary for replication, transcription, and infectious virus assembly. Upon entry into the host cell, to reach the replication stage, the CoV-2 genomic (+) sense ssRNA is used as mRNA to ultimately produce 16 non-structural proteins (nsps) (nsp1–16), numbered according to their order from the N-terminus to the C-terminus of the ORF 1 polypeptides from two large polyproteins, pp1a (4405 amino acids) and pp1ab (7096 amino acids). These two large polyproteins are processed by the main protease, Mpro [also known as the 3-chymotrypsin-like protease (3CLpro)], and by one or two papain-like proteases (PLpro) and translated from the first ORF (ORF 1a/b) which contains a ribosomal frameshift around the middle (Figure 1). This ribosomal frameshift enables a change in the reading frame to form pp1ab after pp1a translation. Therefore, the proper polyprotein processing is essential for the release and maturation of the 16 nsps and assembly into cytoplasmic, ER membrane-bound multicomponent replicase-transcriptase complex (RTC), which is responsible for directing the replication, transcription, and maturation of the viral genome and subgenomic mRNA. Meanwhile, all the four structural and nine accessory proteins (ORF3A, ORF3B, ORF6, ORF7A, ORF7B, ORF8, ORF9B, ORF9C, and ORF10) are translated from subgenomic RNAs (sgRNAs) produced from (−) sense ssRNA of CoVs [14], which is located on the one-third of the genome near the 3′-terminus [1].

The four main structural proteins are the spike (S), membrane (M), envelope (E), and nucleocapsid (N) proteins which are involved in infectious virus assembly [17,18,19,20]. However, more recently, it has become clear that some CoVs do not require the full ensemble of structural proteins to form a complete, infectious virion, suggesting that some structural proteins might be dispensable or that these CoVs might encode additional proteins with overlapping compensatory functions [21,22,23,24,25,26]. Thus, the vital role of the SARS-CoV-2 3CLpro (3CLpro-CoV2) and PLpro (PLpro-CoV2) in proteolytic cleavage of the large viral polyprotein orf1ab and viral replication has established them as promising drug and vaccine targets in the areas of therapeutic research against COVID-19.

Many reports on the screening of natural compounds against SARS-CoV-2 proteases are available which indicate the promising potency of the natural resources to be harnessed and developed as COVID-19 treatment. Nevertheless, most of compounds were derived from the plants. To our knowledge, the microorganisms-based compounds were also reported to be promising to inhibit the SARS-CoV-2 protease. Nevertheless, the reports remain scattered with no solid conclusion. In the present review, literature reports highlight the structure and function of SARS-CoV-2 proteases in viral replication and infection as the promising target for drug development. A more comprehensive understanding towards the structural and functional determination will allow a comprehensive understanding of the infection mechanism and facilitate the processes that can be exploited for structure-guided drug and vaccine design. Furthermore, the development of fungi, cyanobacteria, and their metabolites and peptides as potential drugs for these two viral proteases are also compiled.

## 2. Structure of 3CLpro

**Figure 2:**
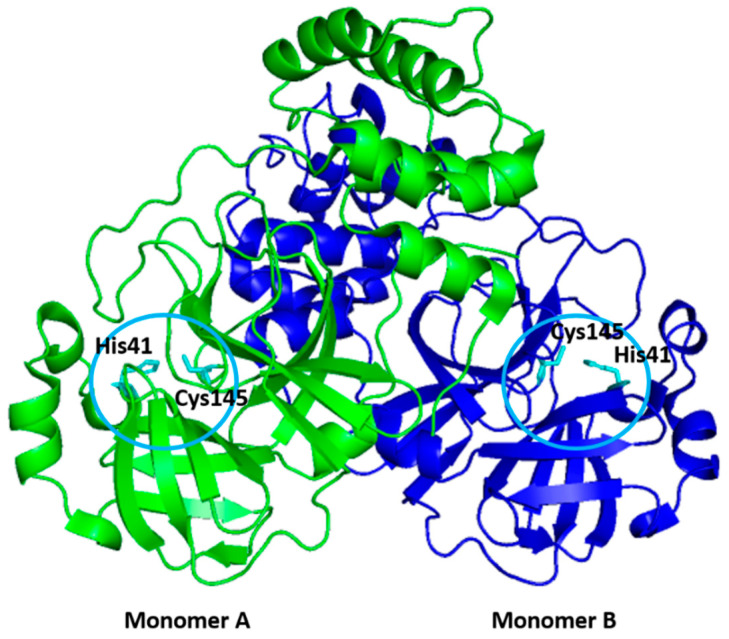
The dimer structure of SARS-CoV-2 3CLpro. Two monomers were indicated by different colour for clarity. The catalytic residues of Cys145 and His41 of each monomer (indicated by circles) are shown in stick (cyan).

**Figure 3:**
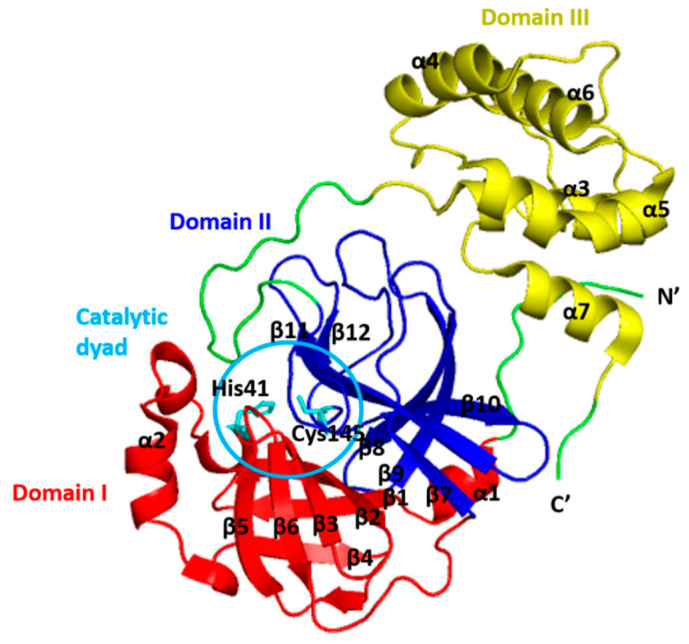
Domain organization of the monomeric structure of the SARS-CoV-2 3CLpro. Domain I (residues 8–101) is coloured in red, domain II (residues 102–184) is coloured in blue and domain III (residues 201–303) is coloured in yellow. The catalytic dyad (His14 and Cys145) of 3CLpro is shown in stick (indicated by circles).

The 3CLpro belongs to the clan PA (family 30) with a catalytic type of mixed cysteine, serine, and threonine [27]. It is a dimer consisting of two monomers that are arranged almost perpendicular to one another (Figure 2), and the individual monomer is enzymatically inactive [28,29,30,31,32]. Each monomer exhibits three-domain structures, as shown in Figure 3 [33,34]. The N-terminal domains (domains I and II) consist of six-stranded antiparallel β-barrel structures, which together resemble the architecture of chymotrypsin and the other 3C proteases found in picornaviruses [35]. It forms a chymotrypsin-like fold between these two domains, hosting the complete catalytic machinery [36]. Therefore, the name of ‘3C-like protease’ refers to the similarities between this protease and the other 3C proteases found in picornaviruses, namely their similar core structural homology and substrate specificities [35].

Unlike other chymotrypsin-like enzymes and many Ser (or Cys) hydrolases, 3CLpro possesses an unconventional Cys catalytic residue. It consists of Cys145 and His41 to form the catalytic Cys-His dyad pair, instead of a canonical Ser(Cys)-His-Asp(Glu) triad in the centre of the cleft between the N-terminal domains [36,37,38,39]. Mechanistic studies suggest an “electrostatic” trigger initiates the acylation step. The Cys145 residue serving as a nucleophile in the enzyme-catalyzed proteolytic reaction, and the imidazole motif of His41 serving as a general base [40]. The cleft accommodates four substrate residues in positions P1′ through P4, and it is flanked by residues from domains I (residues 10–99) and II (residues 100–182) [36]. The active sites of 3CLpro are highly conserved, composing of four subsites (S1′, S1, S2, and S4), and the binding pocket of 3CLpro is mostly hydrophilic, except for the S2 subsite [40].

**Figure 4:**
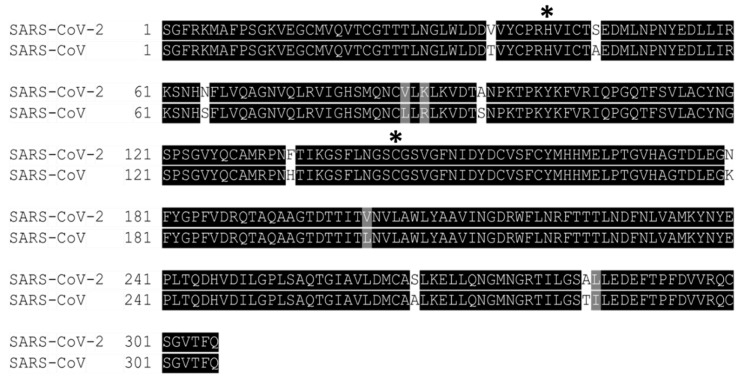
Multiple sequence alignment of 3CLpro between SARS-CoV-2 and SARS-CoV revealed that the sequences shared 96% sequence identity. The asterisk (*) indicates the shared catalytic residues of histidine (H) and cysteine (C). The non-conserved residues were in white or grey colour. Meanwhile, the conserved residues were highlighted in black colour.

**Figure 5:**
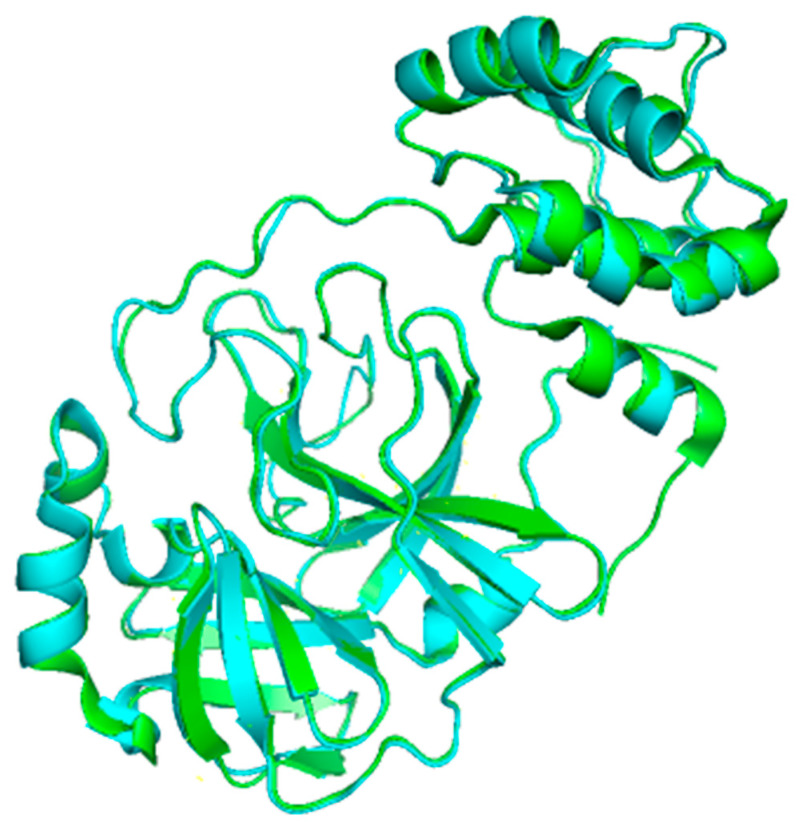
The superimpose of 3CLpro between SARS-CoV-2 (green colour, PDB ID: 7JPY) and SARS-CoV (cyan colour; PDB ID: 2BX4) structures with R.M.S.D value of 0.459 Å. It was visualized with PyMOL.

An extended loop region connects the catalytic domains to the C-terminal domain (domain III). This latter domain (residues 198–303) is composed of a globular cluster of five antiparallel α-helices, which is unique to the CoVs 3CL proteases [41] and is responsible for the enzyme dimerization. A contact interface (∼1394 Å) for the tight dimer is formed predominantly between domain II of monomer A and the NH2-terminal seven residues (N-finger) of monomer B in the 3CLpro-CoV-2 dimeric structure. The dimerization is essential for the enzymatic activity of 3CLpro to assist in the correct orientation of the S1 pocket of the substrate-binding site as the N-finger of each of the two monomers interact with Glu166 of the other monomer [42]. The N-finger is squeezed in between domains II and III of the parent monomer and domain II of the other monomer to reach this interaction site. Sequence alignment revealed that the 3CLpro-CoV-2 shares 96% similarity with SARS, which is highly conservable among CoVs (Figure 4) [33]. Meanwhile, the structure of these proteins (in apo forms) was also found to be very similar as indicated by the low R.M.S.D. value (0.459 Å) from the structural superimposition (Figure 5). Notably, sequence alignment (Figure 4) showed that there are 12 non-conserved residues (out of 306 total residues) in 3CLpro-CoV-2. Nevertheless, mutational works on these residues indicated that none of the mutations on these residues (T35V, A46S, S65N, L86V, R88K, S94A, H134F, K180N, L202V, A267S, T285A, I286L) seriously affected the catalytic activity of 3CLpro-CoV-2. This indicated that the non-conserved residues have no major roles in the enzymatic activity of 3CLpro-CoV-2 [4]. In addition, none of the mutation effected the overall structure of 3CLpro-CoV-2. Interestingly, polar interactions via a hydrogen bond between two Thr285 of each monomer was observed in SARS-CoV, but not in SARS-CoV-2 [40]. The absence of this residue in 3CLpro-CoV-2, nevertheless, has no effect on its dimerization and the activity of this protein.

Among conserved residues, as shown in Figure 4, there are 15 residues (His41, Met49, Gly143, Ser144, His163, His164, Met165, Glu166, Leu167, Asp187, Arg188, Gln189, Thr190, Ala191, Gln192), and these were identified to be responsible for the substrate-binding [30,43]. In addition, 10 residues (Arg4, Ser10, Gly11, Glu14, Asn28, Ser139, Phe140, Ser147, Glu290, Arg298) were found to be responsible for the enzyme dimeric structure based on the previous study of 3CLpro-CoV [44,45,46,47,48,49,50,51].

## 3. Functions of 3CLpro

The 3CLpro, also known as nsp5, is not only the most cysteine protease conserved in structure, but also in its function in all known CoVs [52]. It serves as the main protease for proteolytic processing of the replicase two large polyproteins pp1a and pp1ab (replicase 1ab, ∼790 kDa), and is indispensable for virus replication [5,53,54,55]. The enzyme has a recognition cleavage sequence of Leu-Gln↓Ser-Ala-Gly (↓ marks the cleavage site), in which the Gln forms hydrogen bonds with residues in the S1 subsite and a small amino acid (Ser, Ala, or Gly) occupies the S1′ subsite [40]. The absolute dependence of the virus on the correct function of this protease, along with the absence of a homologous human protease, makes 3CLpro one of the most pursued targets for the development of specific protease inhibitors [33,56].

Prior to processing, 3CLpro is found within a >800 kDa precursor, which is processed into a 150 kDa, comprised of a nsp4 to nsp10/11 precursor [57,58]. The 3CLpro is first automatically cleaved from polyproteins (at its own N-terminal and C-terminal auto processing sites). According to the theory, two 3CLpro proteases anchored to membranes by the transmembrane proteins nsp4 and nsp6 form a dimer and initiate cleavage in trans [59,60,61,62]. Following its maturation cleavage, 3CLpro is believed to target nsp9–10 for processing before moving on to the nsp8–9 and nsp7–8 sites, respectively [63]. Once these sites are processed, the other nsps that 3CLpro is responsible for cleaving are detached from the nsp7–10 site individually. Prior to the final processing of the nsps, one of the intermediate complexes, nsp7+8, performs a significant job catalyzing the cleavage of nsp12, a critical viral polymerase. Therefore, the disruption of the nsp7–nsp8 and nsp8–nsp9 cleavage sites results in nsps loss of virus viability, whereas other sites, such as the nsp9–nsp10 site, can be tolerated with reduced replication in studies involving a mutation of the 3CLpro cleavage sites [64]. Thus, the 3CLpro’s ordered processing may be a unique aspect of viral replication that inhibitors can disrupt.

Other than that, 3CLpro interacts with a variety of different replication complex components. Several studies have found significant intramolecular and intermolecular connections between the 3CLpro and the remainder of the replicase gene, with mutations in the 3CLpro domain as well as mutations in nsp3 and nsp10 all harming 3CLpro activity [58,65,66]. These findings strongly suggest that 3CLpro and other members of the replicase gene have crucial allosteric interactions. Furthermore, multiple temperature-sensitive mutations in the 3CLpro of the mouse hepatitis virus (MHV) and HCoV-OC43 were discovered, which are selected for second-site compensatory alterations that were more than 15 mutations away from the initial mutation site [54,65]. These findings suggest that complex interactions across all three domains of the protease are essential for the structure and function of the enzyme. More research is needed to fully comprehend their role, as they could represent new avenues for proteolytic inhibition.

## 4. Structure of PLpro

**Figure 6:**
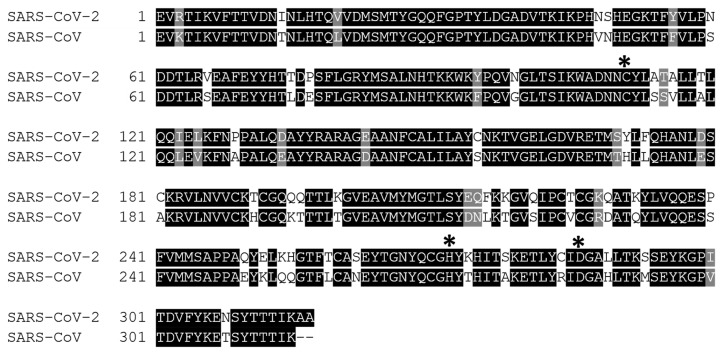
Multiple sequence alignment of PLpro between SARS-CoV-2 and SARS-CoV revealed that the sequences shared 83% sequence identity. The asterisk (*) indicates the shared catalytic residues of cysteine (C), histidine (H) and aspartic acid (D).

**Figure 7:**
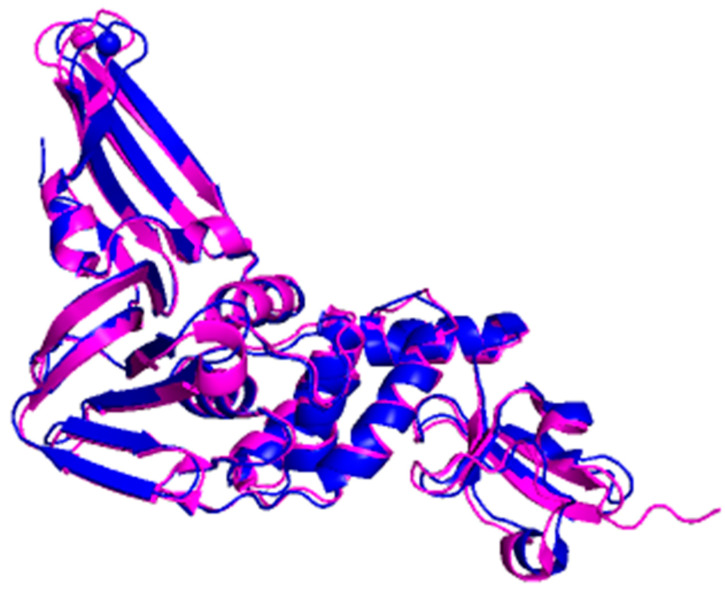
The superimpose of PLpro between SARS-CoV-2 (blue colour, PDB ID: 7D7K) and SARS-CoV (magenta colour; PDB ID: 2FE8) structures with R.M.S.D value of 0.792 Å. It was visualized with PyMOL.

The PLpro belongs to the clan of cysteine proteases CA but is affiliated to the family C16, which contains polyprotein endopeptidases from coronaviruses [27]. It exists as a monomer (about 300 residues) in biological settings and has the USP fold, typical for the ubiquitin-specific proteases (USP) family in humans [67]. The sequence of SARS-CoV-2 PLpro is quite similar with SARS-CoV PLpro as evidence by an 83% sequence identity (Figure 6). In addition, the superimposition between the three-dimensional structure of both enzymes yields 0.792 Å as the value of the R.M.S.D. showing that the proteins are structurally similar (Figure 7).

**Figure 8:**
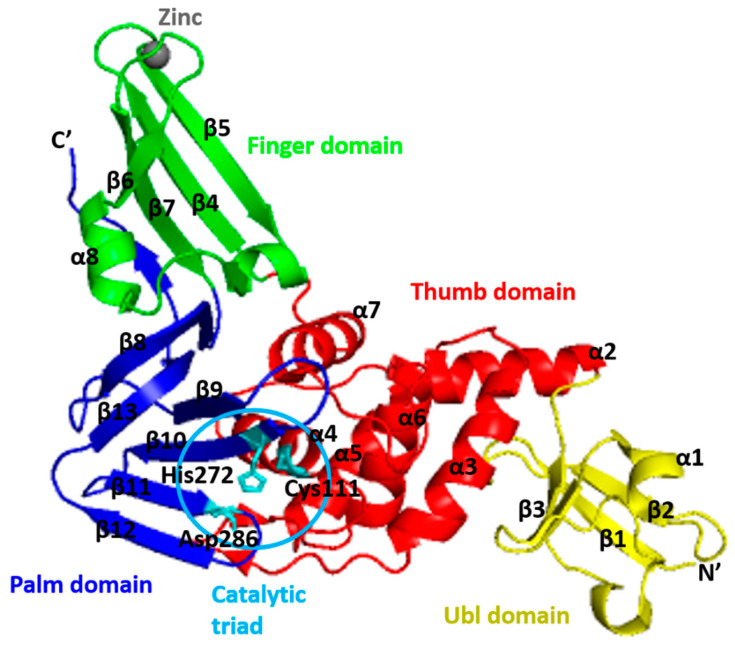
Cartoon representation of the SARS-CoV-2 PLpro. The thumb (residues 61–180) is coloured in red, the palm (residues 239–315) is coloured in blue, the finger (residues 181–238) is coloured in green, and the ubiquitin-like (Ubl) domain (residues 1–60) is coloured in yellow. The interface between the thumb and the palm forms the substrate-binding site leading to the catalytic triad of the active site comprised of Cys111, His272, and Asp286 (shown in stick).

The PLpro-CoV-2 consists of four distinct domains (Figure 8) [68]. The first 60 residues form an independent N-terminal ubiquitin-like (Ubl) domain that is well separated from the other three domains (palm, thumb, and finger domains), which forms a C-terminal ubiquitin-specific protease (Usp) domain [69]. The PLpro Ubl domain has five β-strands, one α-helix, and one 310-helix [14]. It adopts a β-grasp fold which is similar to ubiquitin and Ubl domains of several proteins, including ISG15, yeast yukD, elongin B, tubulin-binding cofactor B, and modifier protein hub 1 [70]. The function of this domain is not well understood, and some studies suggested that it has no effect on the function of PLpro [71]. However, according to Bosken et al. [67], the transposition of Ubl towards the thumb domain resulted in hydrophobic interactions between Pro59 of the Ubl domain and Pro77 and Thr75; Thr75 then interacts with Phe69 of the “ridge” helix and thus, alters the latter residues conformation.

Meanwhile, the C-terminal folds in a canonical thumb–palm–fingers-like structure with the ubiquitin domain anchored to the thumb [72]. In the “open hand” architecture of PLpro, the ubiquitin sits on the “palm” domain and is held in place by the zinc-binding “fingers” domain [73]. The “thumb” domain (residues 107–113, 162–168) is formed by four prominent helices (α4–7), and the palm (residues 269–279) is made up of a six-stranded β-sheet (β8–13) that slopes into the active site, which is housed in a solvent-exposed cleft between the thumb and palm domains [70]. A four-stranded, antiparallel β-sheet makes up the ‘‘fingers’’ domain, and within the fingertip’s region, four cysteine residues coordinate to a zinc ion [74].

The Zn ion is labile and tetrahedrally coordinated by conserved Cys residues (Cys189−X−X−Cys192−Xn−Cys224−X−Cys226). It is essential for the catalysis process because it holds the structural integrity of PLpro-CoV-2 [40,70,75]. Hence, mutation of the zinc-coordinating cysteine caused a significant loss of enzymatic activity, suggesting that the zinc-binding ability is essential for its enzymatic function [75].

The active site of PLpro consists of Cys111, His272, and Asp286, forming the catalytic triad that catalyzed the peptide bond. The nucleophile cysteine (Cys111) is situated at the foot (N-terminus) of α-helix α4 in the thumb domain. The basic histidine (His272) is positioned 3.7 Å from the pros(p)-nitrogen atom of the side chain sulphur atom of Cys111, allowing facile proton transfer [40]. This His272 is located at the foot of the palm domain and adjacent to the flexible β-hairpin loop called the blocking loop two or BL2 (also called the G267–G272 loop) [75,76] or β-turn [77]. The BL2 is a flexible loop that can result in an open or closed conformation [68]. One of the oxygen atoms of the side chain of catalytic aspartic acid (Asp286) is located 2.7 Å from the tele(s)-nitrogen of the catalytic histidine at the foot of the palm domain [78]. Thus, the proposed catalytic cycle involves the catalytic Cys111 as a nucleophile, His272 as a general acid-base, and Asp286 paired with His272 to align and promote deprotonation of Cys111 [40]. Its catalytic domain is also flanked by numerous catalytically active enzymes, transmembrane domains, and domains of unknown function, and the entire nsp3 is localized to the ER membranes where most of the domains reside in the cytosol of the cell [79,80]. Accordingly, the active site residues are located at the interface between the thumb and palm subdomain [81].

## 5. Functions of PLpro

PLpro, also known as the protease domain of nsp3, work together with 3CLpro to generate a functional replicase complex and enable viral spread [82,83]. The enzyme is in nsp3 between the SARS unique domain (SUD/HVR) and a nucleic acid-binding domain (NAB) [14]. It is highly conserved and found in all coronaviruses [84]. The enzyme recognizes a consensus cleavage motif, LXGG (X = any amino acid, L = leucine, and G = glycine) (P4–P1), which is present in two large polyproteins pp1a and pp1ab, corresponding to the P4–P1 substrate positions of cysteine proteases. It will cleave the peptide bonds between nsp1 and nsp2 (LNGG↓AYTR), nsp2 and nsp3 (LKGG↓APTK), and nsp3 and nsp4 (LKGG↓KIVN), with no preference at the P1′ position [14,40]. The cleaving process thus releasing nsp1, nsp2, and nsp3 proteins that will assemble and resulting in the generation of a multifunctional, membrane-associated replicase complex on host membranes, initiating replication and transcription of the viral genome [75,83,85].

The PLpro recognizes and hydrolyzes the cellular proteins of ubiquitin [75] and the Ubiquitine-like (Ubl) protein ISG15 (interferon-induced gene 15) [86,87] as both bearing the LXGG recognition motif at their C-terminus to remove them from host cell proteins [77]. Ubiquitin is a 76-amino-acid protein associated with the regulation of endocytic processes, the cellular response to DNA damage and immunologic processes with the activation of NFkB signalling [81]. The ISG15 modification induced upon viral infection, comprises two fused Ubl-folds structurally resembling diubiquitin [88,89].

Due to the similar recognition motif, PLpro also possesses deubiquitinating and deISGylating capabilities which interfere with critical signaling pathways leading to the expression of type I interferons [40,90]. This interference thus results in an antagonistic effect on the host innate immune response by inhibiting the production of cytokines and chemokines that are responsible for the activation of the host innate immune response against viral infection [78,85,91]. Additionally, PLpro suppresses the NFkB activation [86] and, subsequently, interferes with interferon-β production and suppresses the immune response [92]. Both ubiquitination and ISGylation play important roles in regulating innate immune responses to viral infection. Therefore, it may not be surprising to observe that multiple viruses have evolved different strategies to antagonize these pathways [93]. In conclusion, inhibition of PLpro activity can halt viral replication and disrupt its role in host immune response evasion, making it an excellent antiviral drug target.

## 6. Microorganisms as Sources of Inhibitors Targeting SARS-CoV2 Proteases

The abundance of microorganisms as a living entity on the earth’s surface, which interacts with other organisms, flourishes in the biosphere. Both create an interactive network that constitutes the basis for life on our planet [94,95,96,97]. In recent decades, natural products have been a key source of drug discovery, accounting for 60% of the total market. Interestingly, natural microbial sources account for more than 40% of new drugs discovered since 1980 [98,99]. Until now, the global pharmaceutical industry has relied heavily on natural products from microbial sources to develop novel and effective therapeutics. Natural products derived from microbial sources are considered unique in their chemical diversity in comparison with plant-derived ones. This is due to the tremendous significance of bioactive substances acquired from microorganisms with various biological activities, including antiviral, antibacterial, anticancer, antifungal, and anti-inflammatory. Furthermore, these new substances and novel drugs are cost-effective and highly efficient [100,101]. Since the outbreak of SARS-CoV-2, researchers have studied various microorganisms-based compounds that are promising to inhibit the SARS-CoV-2 proteases (Table 1). Notably, the potency of these compounds is based on in silico studies against either 3CLpro-CoV2 or PLpro-CoV2. Unfortunately, experimental evidence confirming the potency of these compounds against SARS-CoV-2 are so far not available to our knowledge. As shown in Table 1, the promising compounds for 3CLpro-CoV2 or PLpro-CoV2 inhibitors are dominated from *Aspergillus* groups. Antiviral activity of *Aspergillus* groups against some viruses are widely reported [102,103,104,105], nevertheless no report for experimental evidence on the antiviral properties of this group against living SARS-CoV-2 exists. Interestingly, Koehler et al. [106] reported that COVID-19 patients are more susceptible towards invasive pulmonary aspergillosis (IPA). Reports of COVID-19-associated pulmonary aspergillosis have raised concerns about it worsening the disease course of COVID-19 and increasing mortality. This implies that the use of *Aspergillus*-originated compounds for COVID-19 treatments should be in pure form, with no contamination of its cell producers (*Aspergillus*). This is to avoid the possibility of an IPA event during the delivery of compounds.

Further, Table 2 showed the shortlisted microbial natural products with the most promising binding properties against SARS-CoV2 proteases. These compounds were selected based on the best binding energy during the in silico screening. Notably, the compounds shown in Table 2 are not only secondary metabolite compounds, but also some bacteriocins that belong to primary metabolite groups. While the antibiotic is grouped under primary metabolite compounds, bacteriocin bacteriocins are ribosomally synthesized and produced during the primary phase of growth [107].

Overall, the most promising compounds (Table 2) have a wide range of binding energy of −6.9 to −155.3 kcal/mol. Interestingly, plant compounds that were screened against 3CLpro-CoV2 or PLpro-CoV2 have a binding energy ranging from −4.7 to −10.0 (Table 3). This indicated that some microbial natural products are predicted to serve as better inhibitors than the plant compounds. These include citriquinochroman (−14.7 kcal/mol), scedapin C (−10.9 kcal/mol), norquinadoline A (−10.9 kcal/mol), tyrocidine A (−13.1 kcal/mol), gramicidin S (−11.4 kcal/mol), and bacteriocin glycocin F (−155.3 kcal/mol). 

### 6.1. Microbial Natural Products as Potential Inhibitor of 3CLpro-CoV2

The Natural Products Atlas (www.npatlas.org, accessed on 26 June 2021) has created a database of natural products that includes >20,000 compounds from bacteria and fungi and contains referenced data for structure, compound names, source organisms, isolation references, total syntheses, and instances of structural reassignment [122]. To discover potential natural ligands that could block the 3CLpro active site, a study by Sayed et al. [108] utilized this database. The investigation conducted different stages of screening and identified six possible anti-SARS-CoV-2 candidates from the database. The top-scoring compounds in the study are citriquinochroman, holyrine B, proximicin C, pityriacitrin B, (+)-anthrabenzoxocinone, and penimethavone A. The citriquinochroman that can be found in the endophytic fungus *Penicillium citrinum* [123] that got the best hit with the 3CLpro-CoV2 by showing the least binding energy (∆Gaverage = −12.4 kcal/mol), with perfect fitting inside the enzyme active site in the crystallized form. The compounds anchored themselves via a network of H-bond interactions with the reported key binding residues (7 H-bonds) [34]. Despite the flexibility of the enzyme active site, citriquinochroman was able to keep its orientation during the course of simulation with a transient drop in its binding affinity at 3–5.5 ns (∆GVina = −8.9 kcal/mol).

Another study by Rao et al. [109] took the computational approach by screening 100 various small molecules of fungal metabolites from PubChem (https://pubchem.ncbi.nlm.nih.gov accessed on 26 June 2021) using molecular docking and dynamics simulation. The study proposed pyranonigrin A, a secondary fungal metabolite from *Aspergillus niger* [124], to possess potent inhibitory potential against the 3CLpro-CoV2 among these selected fungal metabolites. This fungal metabolite could make seven hydrogen bonds at par with N3 (the positive control compound in the study) and is also predicted to form a covalent bond with 3CLpro-CoV2 making it a promising compound that could be seen for an 3CLpro-CoV2 inhibitor with permanent (irreversible) and strong binding.

Quimque et al. [110] also demonstrated that 14 fungal secondary metabolites displayed a relatively high affinity with 3CLpro-CoV2 with binding energies ranging from −7.9 to –8.9 kcal/mol. Of which, the most notable inhibitor is the anti-HSV metabolite from *Aspergillus terreus* fungus [125], 11a-dehydroxyisoterreulactone A with a −8.9 kcal/mol binding energy. In their in silico study using molecular docking and molecular dynamics simulation, 11a-dehydroxyiso-terreulactone A is found bound to the catalytic residue His41 through pi-pi stacking with the pyranone ring fused in its polycyclic core. The enzyme-ligand complex is further stabilized by H-bonding and pi-alkyl interactions with its methoxyphenyl substituent.

By taking almost similar computational approaches, a study by El-Hawary et al. [111] identified aspergillide B1 and 3a-hydroxy-3, and 5-dihydromonacolin L, and noted that they can interact with catalytic dyad (His41 and Cys145) amino acid residues of 3CLpro-CoV2. These metabolites are produced by the endophytic fungus, *Aspergillus terreus*, which is a similar microbe to Quimque et al. [110]. The molecular docking studies by [111] have proposed that both metabolites showed the highest binding energy scores (aspergillide B = −9.473 kcal/mol) and (3a-hydroxy-3,5-dihydromonacolin L = −9.386 kcal/mol) towards 3CLpro-CoV2. Thus, making them potent as COVID-19 drug candidates.

Sterenin M, an isoprenylated depside, which was first isolated in from a culture of the mushroom *Stereum hirsutumcan* in the year of 2014 [126], also interacted with the 3CLpro-CoV2 catalytic dyad as reported by Prajapati et al. [112]. By screening more than 1800 chemically diverse and therapeutically important secondary metabolites available in the Medicinal Fungi Secondary Metabolites and Therapeutics (MeFSAT) database, their study found five fungal metabolites are having this interaction. However, only sterenin M is making hydrogen bonds with the key amino acids of 3CLpro-CoV2 (Gly143, His163, Phe140, and Glu166).

Other potential inhibitors that interacted with catalytic dyad amino acid residues of 3CLpro-CoV2 are stigmasterol, chondrillasterol, and hexadecnoic acid, compounds that were isolated from the crude extracts *Bacillus* species [113]. They bind in the substrate-binding pocket of 3CLpro-CoV2. Among the top three docking hits, hexadecanoic acid was found to be the most promising anti-COVID-19 lead against the main protease after further evaluation using 50 ns molecular dynamic simulation and MMPB-GBSA.

Not only compounds from the *Bacillus* species, metabolites from cyanobacteria also showed potential as 3CLpro-CoV2 inhibitors as found in the study by Naidoo et al. [114]. By employing an in silico molecular interaction-based approach, metabolites cylindrospermopsin, deoxycylindrospermopsin, carrageenan, cryptophycin 52, eucapsitrione, tjipanazole, tolyporphin, and apratoxin A exhibited promising inhibitory potential against this protease. Among the 23 chemically diverse biologically active metabolites from the cyanobacteria, the cyanotoxin, deoxycylindrospermopsin originally isolated from *Cylindrospermopsis* sp. displayed the most promising binding efficiency (−8.6 ± 0.02 kcal/mol) and interacted with the important residues at the active binding pocket of 3CLpro-CoV2. This includes the binding with Glu166 that is very important for the dimeric structure of this protease [42].

Apart from that, antimicrobial peptides (AMPs) produced by different organisms also have been considered as potential antiviral drugs against COVID-19. Many microorganisms produce these peptides as their innate immune response component against invading pathogens [115,127,128]. They also have been reported to exert a powerful antimicrobial effect on the membrane of different pathogenic microorganisms including bacteria, fungi, and viruses [127,129]. Due to this potential, Balmeh et al. [115] utilized StraPep (http://isyslab.info/StraPep/ accessed on 15 July 2021) and PhytAMP (http://phytamp.hammamilab.org/main.php accessed on 15 July 2021) databases to figure out AMPs. Among 500 bio-peptides that were analyzed, bacteriocin glycocin F from the *Lactobacillus* plantarum showed the highest binding affinity towards 3CLpro-CoV2 with 155.3 ± 7.5 kcal/mol. The obtained score was calculated as a high score in the HADDOCK molecular docking scoring system, thus, showing the potential of bacteriocin glycocin F as a 3CLpro-CoV2 inhibitor.

### 6.2. Microbial Natural Products as a Potential Inhibitor of PLpro-CoV2

For millennia, medicinal fungi have been used to treat human illnesses in traditional remedies. Fungi are abundant in secondary metabolites, which provide a valuable and diverse chemical resource of natural products with potential bioactivity [112]. Fungal metabolites have not just been screened for potential inhibitor against 3CLpro-CoV2 [109], but also for PLpro-CoV2. A study conducted by [116] retrieved 100 naturally occurring secondary fungal secondary metabolites with aromatic moiety. Its aim was to identify the analogue of GRL0617, the naphthalene-based compounds that can effectively inhibit PLpro-CoV2 [69]. Interestingly, six hits were found that can interact with the Tyr268 residue of PLpro-CoV2 and the lead fungal metabolite identified is fonsecin. Fonsecin is a naphthopyrone pigment isolated from a mutant of *Aspergillus fonsecaeus* [130] and had a binding energy at par with GRL0617. Thus, it is believed that this fungal metabolite can inhibit PLpro-CoV2 as deduced using docking and molecular dynamics.

Quimque et al. [110] also discovered the potential of secondary metabolites from fungi as potential drug prototypes against the SARS-CoV-2 virus. All the screened fungal secondary metabolites were selected with profound antiviral activity on a range of known pathogenic viruses such as the human immunodeficiency virus (HIV), influenza virus, herpes simplex virus (HSV), and hepatitis C virus (HCV). The study disclosed that the in silico aided discovery of two fumiquinazoline marine alkaloids of scedapin C (−10.9 kcal/mol) and norquinadoline A (−10.9 kcal/mol) exhibiting strong in silico binding activity against PLpro-CoV2. Scedapin C was isolated from the marine-derived fungus *Scedosporium apiospermum* F41−1 and displayed significant antiviral activity against hepatitis C [131]. It was noted to be bound to the putative binding site of PLpro-CoV2 through H-bonding with two ketones of the quinazolinedione core against Arg712 during the post-dock analysis. On the other hand, norquinadoline A that was isolated from the mangrove-derived fungus *Cladosporium* sp. PJX-41 showed activity against influenza A (H1N1) [132]. It was tightly nestled to the PLpro-CoV2′s binding site, stabilized mostly by van der Waals forces.

Another study employing an in silico molecular interaction-based approach, has screened 23 selected biologically active cyanobacterial metabolites [114]. Metabolites cylindrospermopsin, deoxycylindrospermopsin, carrageenan, cryptophycin 52, eucapsitrione, tjipanazole, tolyporphin, and apratoxin A not only exhibited as potential inhibitors against 3CLpro-CoV2, but also PLpro-CoV2. Subsequent analysis of physicochemical features, potential toxicity, molecular dynamics simulations, and MM-PBSA energy scoring function finally established deoxycylindrospermopsin as the most promising candidate for PLpro-CoV2. It displayed the highest binding energy scores of 7.9 ± 0.04 kcal/mol and interacted with the amino acid residues Lys157, Gly163, Asp164, Arg166, Ala246, Tyr264, Thr301, and Asp302 at the active binding pocket of PLpro [133].

Apart from that, a study by Bansal et al. [117] has utilized non-ribosomal peptide synthetases (NRPs) from the PubChem database as potential inhibitors for PLpro-CoV2. Through the molecular docking, 21 pharmacologically active NRPs from marine microbes showed strong interaction with the protease. Out of the 21 screened ligands, the two peptides produced by *Bacillus brevis* [134,135], tyrocidine A and gramicidin S, showed the highest binding affinities with −13.1 kcal/mol and −11.4 kcal/mol, respectively, for PLpro-CoV2. Tyrocidine A exhibited hydrogen bonding with Lys105, and established π-alkyl interactions with Leu162 and Met208. It also showed π-cation interactions with Lys157, Asp164, and Glu167 residues. Meanwhile, gramicidin S formed four hydrogen bonds with Lys157, Glu161, Lys105, and Asp108, and π-cation and anion interactions with Lys157, Asp164, and Glu167 residues. The ligand also formed alkyl and π-alkyl interactions with Met208 and Pro247 residues. The docking results thereby further suggesting that these peptides might be used in inhibiting PLpro-CoV2.

## 7. Possibility of Inhibitors Targeting HIV Protease for SARS-CoV2 Proteases

It is interesting that the strategy of inhibition of proteases is also applied for the discovery of drugs targeting the human immunodeficiency viruses (HIV), the retrovirus that causes human immunodeficiency virus infection and acquired immunodeficiency syndrome (HIV/AIDS). This virus is equipped with an aspartic protease with the catalytic tried active sites of Asp-Thr-Gly (Asp25, Thr26, and Gly27). This protease is essential for virus replication, particularly to hydrolyze peptide bonds on the Gag-Pol polyproteins at nine specific sites, processing the resulting subunits into mature, fully functional proteins. These cleaved proteins, including reverse transcriptase, integrase, and RNaseH, are encoded by the coding region components necessary for viral replication [136].

To note, significant differences were observed on the genomic structure and proteases of SARS-CoV2 and HIV. Nevertheless, the recent reports on the promising use of lopinavir/ritonavir, approved anti-HIV drugs, to inhibit SARS-CoV2 [137,138] might be indicating that the repurpose strategy of HIV proteases for SARS-CoV-2 protease was apparently possible. Thran et al. [139] reported that lopinavir, darunavir, atazanavir, remdesivir, and tipranavir demonstrated the promising inhibitory properties against 3CLpro-CoV2 with the binding energy ranging from −8.4 to −9.2 kcal/mol. These compounds are the FDA-approved drugs of HIV. Similarly, Raphael and Shanmughan [140] also examined the HIV-protease inhibitors of atazanavir, darunavir, fosamprenavir, saquinavir, lopinavir, ritonavir, nelfinavir, and indinavir against 3CLpro-CoV2. The result showed that the binding energy ranged from −6.4 to −9.0 kcal/mol. Bolcato et al. [141] also demonstrated that lopinavir, ritonavir, and nelfinavir indeed were able to bind and lock the catalytic site of the 3CLpro-CoV2. Experimental evidence performed by Mahdi et al. [142] showed that lopinavir, ritonavir, darunavir, saquinavir, and atazanavir could inhibit the viral protease in cell culture, albeit in concentrations much higher than their achievable plasma levels, given their current drug formulations

It is interesting to note that Table 2 showed that some microbial natural products have better binding energy to 3CLpro-CoV2 than these FDA-approved drugs of HIV. In particular, aspergillide B1 (−9.47 kcal/mol), 3α-Hydroxy-3,5-dihydromonacolin L (−9.39 kcal/mol), citriquinochroman (−14.7 kcal/mol), scedapin C (−10.9 kcal/mol), norquinadoline A (−10.9 kcal/mol), tyrocidine A (−13.1 kcal/mol), gramicidin S (−11.4 kcal/mol), and bacteriocin glycocin F (−155.3 kcal/mol) have better binding energy than the HIV protease reported. This implied that these microbial compounds possibly bind and inhibit the SARS-CoV-2 proteases better than the HIV protease inhibitors. To note, cell culture works reported by Mahdi et al. [142] indicated that complete inhibition of the virus required a high concentration of HIV protease inhibitors. This limits the clinical potential of the inhibitors for SARS-CoV-2 drugs. The high concentration requirement is likely associated to weak binding affinity of HIV protease inhibitors toward SARS-CoV-2 proteases, particularly 3CLpro-CoV2. As some microbial compounds displayed better binding energy to 3CLpro-CoV2 than HIV protease inhibitors, this might imply that the microbial compounds are able to inhibit 3CLpro-CoV2 protease at a lower concentration than the HIV protease inhibitors. The weak binding affinity of HIV protease inhibitors against 3CLpro-CoV2 is understandable as the compounds were initially designed to block HIV protease active sites. Structurally, HIV protease and 3CLpro-CoV2 are significantly different. In addition, the nature of the HIV protease as an aspartic protease is indeed different to the 3CLpro-CoV2 as a cysteine protease. Accordingly, the microbial natural products that are screened directly against 3CLpro-CoV2 might likely perform better than the HIV protease inhibitors. To note, there is no study on the binding assay of HIV protease inhibitors against PLpro-CoV2 due to extreme structural differences between both proteases. 

Altogether, the use of microbial compounds is therefore advantageous as the compounds can inhibit both SARS-CoV-2 proteases. Nevertheless, further experimental confirmation on inhibitory activities of microbial-based compounds against SARS-CoV-2 proteases remains to be conducted. For this purpose, our previous success in the production of recombinant 3CLpro-CoV2 and PLpro-CoV2 [143] is advantageous for further compound testing.

## 8. Conclusions

By a million confirmed cases worldwide, the SARS-CoV-2 pandemic crisis has caused mounting mortality rates and economic devastation that have made many people despair. In these few decades, this is the third pandemic caused by CoVs, after SARS and MERS, but to no avail, there are still no confirmed drugs for treatment. Thus, it has become challenging for researchers to formulate effective therapeutics that take years of investigation and cost billions of dollars. Targeting 3CLPro and PLpro has become the aim of many studies to find molecules of compounds that can become promising inhibitors to stop viral replication. A comprehensive understanding on the structure and function of SARS-CoV-2 proteases is undoubtedly essential to serve as platform for the discovery of promising inhibitors. Among many natural compounds, microorganism-based compounds were also reported to exhibit inhibitory properties toward SARS-CoV-2′s proteases. As the microorganisms are relatively easily cultivated and harnessed to get the compounds, the use of microorganism-based compounds to target SARS-CoV-2 proteases is therefore advantageous. In addition, comparison with the HIV protease inhibitors indicated that microbial-based compounds apparently have better inhibition properties against the SARS-CoV-2 protease. This should contribute to a speedy discovery of potential treatments for COVID-19.

## Figures and Tables

**Table 1:** List of the potential inhibitors from microbial sources for SARS-CoV-2 proteases.

Compound/Peptide	Type	Organism	Ref.
**Potential Inhibitor for 3CLpro-CoV2**			
Citriquinochroman	Hydroquinolones	*Penicillium citrinum*	[108]
Holyrine B	Indolocarbazole	*Streptococcus mutans*	
Proximicin C	Furan analogue of netropsin	*Verrucosispora maris*	
Pityriacitrin B	Ultraviolet-absorbing indole alkaloid	*Malassezia furfur*	
(+)-Anthrabenzoxocinone	Aromatic polyketide	*Streptomyces* sp.	
Penimethavone A	Flavone	*Penicillium chrysogenum*	
Pyranonigrin A	Pyranopyrroles	*Aspergillus niger*	[109]
Altertoxin V	Perylene quinone	*Alternaria* spp.	[110]
Altertoxin II	Perylene quinone	*Alternaria* spp.	
Penicillixanthone A	Xanthone dimer	*Aspergillus fumigates*	
Cytochalasin Z8	Cytochalasan alkaloid	*Spicaria elegans*	
Chloropupukeanolide A	Spiroketal peroxides	*Pestalotiopsis fici*	
Phomasetin	Alkaloids	*Phoma* sp.	
Isochaetochromin D1	Polyketide	*Fusarium* sp.	
Aspergilol H (R)	Anthraquinones	*Aspergillus versicolor*	
Aspergilol H (S)	Anthraquinones	*Aspergillus versicolor*	
11a-Dehydroxyiso-terreulactone A	Terpenoid	*Aspergillus terreus* SCSGAF0162	
Arisugacin A	Aromatic ether organic heterotetracyclic	*Penicillium* sp.	
Aspernolide A	Butyrolactone secondary metabolite	*Cladosporium cladosporioides*	
Rhodatin	Meroterpenoid	*Rhodotus palmatus*	
Scedapin C	Fumiquinazoline alkaloids	*Scedosporium apiospermum*	
Scequinadoline A	Fumiquinozalines	*Dichotomomyces cejpii*	
14S-Oxoglyantrypine	Indole alkaloids containing pyrazinoquinazoline-derivative framework	*Cladosporium* sp. PJX-41	
Deoxynortryptoquivaline	Alkaloid	*Cladosporium* sp. PJX-41	
Quinadoline B	Alkaloid	*Cladosporium* sp. PJX-41	
Norquinadoline A	Fumiquinazoline alkaloids	*Cladosporium* sp. PJX-41	
Asperterrestide A (S)	Cyclic tetrapeptide	*Aspergillus terreus* SCSGAF0162	
Asperterrestide A (R)	Cyclic tetrapeptide	*Aspergillus terreus* SCSGAF0162	
Rubrolide S	Rubrolide	*Aspergillus terreus* OUCMDZ-1925	
Isoaspulvinone	Aspulvinone	*Aspergillus terreus*	
Aspergilide B1	Butenolide	*Aspergillus ostianus strain 01F313*	[111]
3α-hydroxy-3,5-dihydromonacolin L	Polyketide	*Aspergillus terreus*	
2-cyclohexyl-∼{*N*}-pyridin-3-yl-ethanamide	Non-polymer	*Aspergillus terreus*	
Sulochrin	Benzophenone	*Aspergillus terreus, Aureobasidium*	
Emodin	Trihydroxyanthraquinone	*Aspergillus ochraceus, Aspergillus wentii, Aspergillus terreus*	
Reticulol (6-demethylkigelin)	Isocoumarin	*Aspergillus terreus*	
Aspergiketal	Spiroketal	*Aspergillus terreus*	
Terrelactone A	Butyrolactone	*Aspergillus terreus*	
Dihydrocitrinone	Isocoumarin	*Aspergillus terreus*	
4-Hydroxykigelin	Isocoumarin	*Aspergillus terreus*	
Terreic acid	Quinone epoxide	*Aspergillus terreus*	
Flavipin	Polyketide	*Aspergillus flavipes, Aspergillus terreus,* *Aspergillus fumigatus*	
(3S,6S)-Terramide A	Diketopiperazine alkaloid	*Aspergillus terreus, Aspergillus flavus*	
3-Methylorsellinic acid	Phenolic acid	*Aspergillus terreus*	
Terremutin hydrate	Dihydrotoluquinones	*Aspergillus terreus*	
Poh 3	Class I hydrophobins	*Pleurotus ostreatus*	[112]
Epi-phelligrin A	Phenylpropanoids and polyketides	*Sanghuangporus baumii*	
Sterenin M	Isoprenylated depside	*Stereum hirsutum*	
Termitomycamide B	Indoles	*Termitomyces titanicus*	
Enokipodin D	Quinones	*Flammulina velutipes*	
Chondrillasterol	Steroid	*Paenibacillus dendridiformis*	[113]
Cholestan	Sterol lipids	*Paenibacillus dendridiformis*	
Trifluoroacetic acid	Organofluorine	*Paenibacillus dendridiformis*	
Octadeccenoic-acid	Glycerol ester	*Bacillus subtilis*	
Stigmasterol	Stigmastanes	*Paenibacillus dendridiformis*	
Octadecenoic acid	Monocarboxylic acid	*Paenibacillus dendridiformis*	
Hexadecanoic acid	Fatty acid	*Bacillus subtilis*	
Apratoxin A	Cyclodepsipeptide	*Cyanobacterial lyngbya* spp.	[114]
Carrageenan	Sulfated polysaccharides	*Chondrus crispus*	
Cryptophycin 52	Dioxadiazacyclohexadecenetetrone cytotoxins	*Nostoc cyanobacteria*	
Cylindrospermopsin	Cyclic guanidine alkaloid	*Cylindrospermopsis, Aphanizomenon,* *Anabaena, Lyngbya, Umezakia, and Raphidiopsis*	
Deoxycylindrospermopsin	Triazaacenaphthylene	*Cylindrospermopsis raciborskii*	
Eucapsitrione	Anthraquinone	*Cyanobacterium eucapsis* sp.	
Tjipanazole A1	Alkaloids	*Tolypothrix tjipanasensis*	
Tolyporphin	Tetrapyrroles	*Tolypothrix nodosa*	
Bacteriocin glycocin F	Peptide	*Lactobacillus plantarum*	[115]
Cathelidicin-6	Peptide	*Bos taurus*	
Subtilosin-A	Peptide	*Bacillus subtilis*	
Bacteriocin PlnK	Peptide	*Lactobacillus plantarum*	
Moronecidin	Peptide	*Morone saxatilis*	
Bacteriocin lactococcin-G subunit beta	Peptide	*Lactococcus lactis subsp. Lactis*	
Crotamine Ile-19	Peptide	*Crotalus durissus ruruima*	
Bacteriocin leucocin-A	Peptide	*Leuconostoc gelidum*	
M-zodatoxin-Lt2a	Peptide	*Lachesana tarabaevi*	
Polyphemusin-1	Peptide	*Limulus polyphemus*	
Corticostatin-related peptide RK-1	Peptide	*Oryctolagus cuniculus*	
**Potential Inhibitor for PLpro-CoV2**			
Fonsecin	Naphtho-gamma-pyrone	*Aspergillus fonsecaeus*	[116]
Pyranonigrin-B	Pyranopyrroles	*Aspergillus niger* LL-LV3020	
Nigerloxin	Benzoic acid	*Aspergillus niger*	
Flaviolin	Naphthoquinones	*Aspergillus* sp.	
Tensidol A	Furopyrrols	*Aspergillus niger*	
Ochratoxin Beta	Fungal metabolites	*Aspergillus ochraceus*	
Altertoxin V	Perylene quinone	*Alternaria tenuissima*	[110]
Altertoxin II	Perylene quinone	*Alternaria tenuissima*	
Penicillixanthone A	Xanthone dimer	*Aspergillus fumigates*	
Cytochalasin Z8	Cytochalasan alkaloid	*Spicaria elegans*	
Stachybotrosin D	Alcoholic O-sulfation	*Stachybotrys chartarum*	
Chloropupukeanolide A	Spiroketal peroxides	*Pestalotiopsis fici*	
Phomasetin	Alkaloids	*Phoma* sp.	
Isochaetochromin D1	Polyketide	*Fusarium* sp.	
Aspergilol H (R)	Anthraquinones	*Aspergillus versicolor*	
Aspergilol H (S)	Anthraquinones	*Aspergillus versicolor*	
11a-Dehydroxyiso-terreulactone A	Terpenoid	*Aspergillus terreus* SCSGAF0162	
Arisugacin A	Aromatic ether organic heterotetracyclic	*Penicillium* sp.	
Aspernolide A	Butyrolactone secondary metabolite	*Cladosporium cladosporioides*	
Rhodatin	Meroterpenoid	*Rhodotus palmatus*	
Scedapin C	Fumiquinazoline alkaloids	*Scedosporium apiospermum*	
Scequinadoline A	Fumiquinozalines	*Dichotomomyces cejpii*	
14S-Oxoglyantrypine	Indole alkaloid	*Cladosporium* sp. PJX-41	
Deoxynortryptoquivaline	Quinazoline alkaloid	*Aspergillus clavatus*	
Quinadoline B	Alkaloid	*Cladosporium* sp. PJX-41	
Norquinadoline A	Fumiquinazoline alkaloids	*Cladosporium* sp. PJX-41	
Asperterrestide A (S)	Cyclic tetrapeptide	*Aspergillus terreus* SCSGAF0162	
Asperterrestide A (R)	Cyclic tetrapeptide	*Aspergillus terreus* SCSGAF0162	
Rubrolide S	Rubrolide	*Aspergillus terreus* OUCMDZ-1925	
Isoaspulvinone	Aspulvinone	*Aspergillus terreus*	
Fulvic acid	Organic acid	Many microorganisms	
Cryptophycin 1	Peptolides	*Nostoc* sp. ATCC 53789, *Nostoc* sp. GSV 224.	[114]
Cryptophycin 52	Peptolides	*Nostoc* sp.	
Deoxycylindrospermopsin	Triazaacenaphthylene	*Cylindrospermopsis raciborskii*	
Fijimycin A	Cyclic depsipeptide	*Streptomyces* sp.	[117]
Kocurin	Thiazolyl peptide	*Kocuria palustris*	
Cyclosporin A	Cyclic non-ribosomal peptides	*Beauveria nivea*	
Dactinomycin	Chromopeptide antineoplastic antibiotic	*Streptomyces parvulus*	
Daptomycin	Lipopeptide antibiotic	*Streptomyces roseosporus*	
Emericellamides A	Cyclodepsipeptide	*Emericella* sp.	
Trichoderin	Mycobacterial aminolipopeptide	*Trichoderma* sp.	
Marthiapeptide	Polythiazole cyclopeptide	*Brevibacillus* sp.	
Leodoglucomide	Microbial non-ribosomal peptide	*Bacillus licheniformis*	
Unguisin	Cyclic heptapeptide	*Emericella unguis*	
Lajolamycin	Microbial non-ribosomal peptide	*Streptomyces nodosus*	
Brunsvicamide A	Cyclic peptide	*Tychonema* sp.	
Tyrocidine A	Cyclic decapeptide	*Bacillus brevis*	
11-O-methylpseurotin A	Fungal metabolite	*Aspergillus fumigatus*	
Lobocyclamide B	Cyclododecapeptide	*Lyngbya confervoides*	
Ngercheumicin I	Cyclic depsipeptide	*Photobacterium* sp.	
Nocathiacins I	Cyclic thiazolyl peptides	*Nocardia* sp.	
Solonamide A	Non-ribosomal depsipeptide	*Photobacterium* sp.	
Thiocoraline	Cyclic depsipeptide	*Micromonospora. I.*	
Gramicidin S	Cyclic decapeptide	*Bacillus brevis*	

**Table 2:** List of the highest potential inhibitors from microbial sources for SARS-CoV-2 proteases with their binding energy.

Structure	Compound/Peptide	Method Analysis	Binding Energy (kcal/mol)	Ref.
**Potential Inhibitor for 3CLpro-CoV2**				
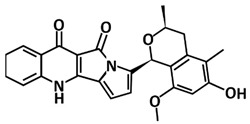	Citriquinochroman	Pharmacophore-based virtual screening using Pharmit molecular docking Autodock Vina	−14.7	[108]
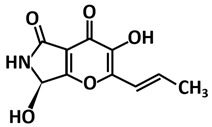	Pyranonigrin A	Molecular docking Autodock Vina, molecular dynamics using GROMACS 2019, ADMET analysis pkCSM–pharmacokinetics server	−7.3	[109]
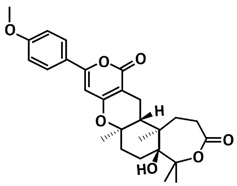	11a-dehydroxyisoterreulactone A	Molecular docking using UCSF Chimera, molecular dynamics using Amber 18, computational prediction of the absorption, distribution, metabolism, and excretion (ADME) properties using SwissADME software	−8.9	[110]
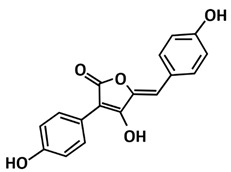	Aspergillide B1	Molecular docking using OpenEye’s FRED	−9.473	[111]
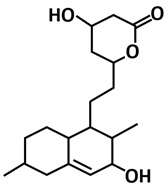	3α-Hydroxy-3,5-dihydromonacolin L	Molecular docking using OpenEye’s FRED	−9.386	[111]
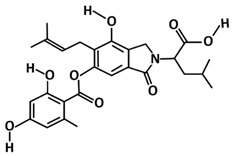	Sterenin M	Molecular docking using Glide, ADMET analysis pkCSM–pharmacokinetics server, molecular dynamics using Desmond	−8.431	[112]
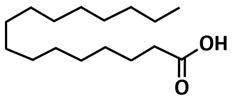	Hexadecanoic acid	Molecular docking using AutoDock, molecular dynamics using Amber 18	−6.9	[113]
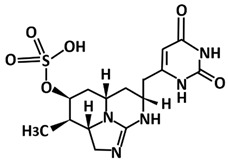	Deoxycylindrospermopsin	Molecular docking using AutoDock Vina, molecular dynamics using GROMACS 2019	−8.6	[114]
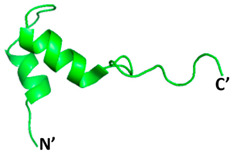 PDB ID: 2KUY	Bacteriocin glycocin F	Molecular docking using UCSF Chimera, molecular dynamics using GROMACS	−155.3	[115]
**Potential Inhibitor for PLpro-CoV2**				
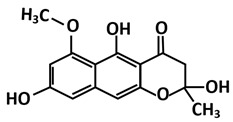	Fonsecin	Molecular docking using AutoDock Vina, ADMET analysis pkCSM–pharmacokinetics server	−7.25	[116]
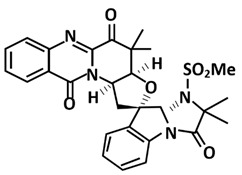	Scedapin C	Molecular docking using UCSF Chimera, molecular dynamics using Amber 18, computational prediction of the absorption, distribution, metabolism, and excretion (ADME) properties using SwissADME software	−10.9	[110]
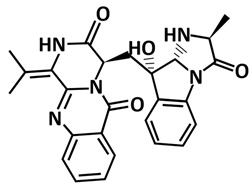	Norquinadoline A	Molecular docking using UCSF chimera, molecular dynamics using Amber 18, computational prediction of the absorption, distribution, metabolism, and excretion (ADME) properties using SwissADME software	−10.9	[110]
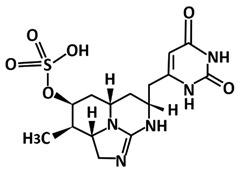	Deoxycylindrospermopsin	Molecular docking using AutoDock Vina, molecular dynamics using GROMACS 2019	−7.9	[114]
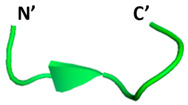 PDB ID: 4M6E	Tyrocidine A	Molecular docking using AutoDock Vina	−13.1	[117]
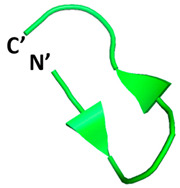 PDB ID: 1TK2	Gramicidin S	Molecular docking using AutoDock Vina	−11.4	[117]

Note: The structure of peptide-based compounds are displayed in their three-dimensional structure.

**Table 3:** Plant compounds thar are screened against SARS-CoV-2 proteases.

Compound	Type	Binding Energy (kcal/mol)	Ref.
**Plant Compounds Screened Against 3CLpro-CoV2**			
10-hydroxyusambarensine	Indole alkaloid	−10.0	[118]
Cryptoquindoline	Cryptolepine	−9.7	
Cryptospirolepine	Cryptolepine	−9.1	
Chrysopentamine	Indole alkaloid	−8.6	
Isocryptolepine	Cryptolepine	−8.5	
Strychnopentamine	Indole akaloid	−8.2	
Isostrychnopentamine	Indole akaloid	−8.1	
Normelicopicine	Acridone	−8.1	
Jozipeltine A	Naphthoisoquinoline	−8.0	
5′-*O*-demethyl-dioncophylline A	Naphthoisoquinoline	−8.0	
Dioncophylline C	Naphthoisoquinoline	−7.9	
Dioncopeltine A	Naphthoisoquinolines	−7.8	
Liriodenine	Indole alkaloid	−7.6	
5,6-dihydronitidine	Furoquinoline	−7.6	
Hydroxycryptolepine	Cryptolepine	−7.6	
Cryptoheptine	Cryptolepine	−7.6	
Annonidine F	Indole alkaloid	−7.5	
Ancistrotanzanine C	Naphthoisoquinoline	−7.5	
Fagaronine	Indole alkaloid	−7.4	
Alstonine	Indole alkaloid	−7.4	
Curcumin	Curcuminoid	−6.04	[119]
Bisdemethoxycurcumin	Curcuminoid	−7.3	
Demethoxycurcumin	Curcuminoid	−7.02	
Scutellarin	Flavanoid	−7.13	
Quercetin	Flavanoid	−6.58	
Myricetin	Flavanoid	−6.15	
Bergapten	5-methoxypsoralen	5.98	
Isoflavone	Flavanoid	5.69	
Spicatolignan	Lignan	−6.7403	[120]
Vanillic acid	Benzenoid	−4.8624	
Ferulic acid	Hydroxycinnamic acid	−5.3292	
Pinoresinol	Furanoid ligan	−6.463	
Sesamolin	Lignan	−6.829	
Sesamin	Lignan	−6.7157	
Hydroxymatairesinol	Lignan	−7.4674	
Saikosaponin D	Saikosaponin	−8.9	[121]
Saikosaponin E	Saikosaponin	−8.9	
Myricetin	Flavanoid	−8.9	
Theaflavin	Catechin	−8.6	
Glycyrrhizin	Triterpenoid saponin	−8.7	
**Plant compounds screened against PLpro-CoV2**			
Hydroxymatairesinol	Lignan	−7.2085	[120]
Spicatolignan	Lignan	−6.6183	
Vanillic acid	Benzenoid	−4.6805	
Ferulic acid	Hydroxycinnamic acid	−4.8177	
Pinoresinol	Furanoid ligan	−6.5131	
Sesamolin	Lignan	−6.454	
Sesamin	Lignan	−6.5524	
Amentoflavone	Biflavonoid	−9.2	[121]
Glycyrrhizin	Triterpenoid saponin	−9.6	
Theaflavin	Biflavonoid	−9.1	
Chrysin-7-*O*-glucuronide	Flavonoid	−8.8	
Isoquercitrin	Flavonoid-3-*O*-glycoside	−8.5	

## References

[1] Zhou P., Yang X.L., Wang X.G., Hu B., Zhang L., Zhang W., Si H.R., Zhu Y., Li B., Huang C.L. (2020). A pneumonia outbreak associated with a new coronavirus of probable bat origin. Nature.

[2] Wu F., Zhao S., Yu B., Chen Y.M., Wang W., Song Z.G., Hu Y., Tao Z.W., Tian J.H., Pei Y.Y. (2020). A new coronavirus associated with human respiratory disease in China. Nature.

[3] Gorbalenya A.E., Haagmans B.L., Sola I. (2020). The species severe acute respiratory syndrome-related coronavirus: Classifying 2019-ncov and naming it SARS-CoV-2. Nat. Microbiol..

[4] Goyal B., Goyal D. (2020). Targeting the dimerization of the main protease of coronaviruses: A potential broad-spectrum therapeutic strategy. ACS Comb. Sci..

[5] Fehr A.R., Perlman S. (2015). Coronaviruses: An overview of their replication and pathogenesis. Methods Mol. Biol..

[6] Woo P.C., Lau S.K., Lam C.S., Lau C.C., Tsang A.K., Lau J.H., Bai R., Teng J.L., Tsang C.C., Wang M. (2012). Discovery of seven novel mammalian and avian coronaviruses in the genus deltacoronavirus supports bat coronaviruses as the gene source of alphacoronavirus and betacoronavirus and avian coronaviruses as the gene source of gammacoronavirus and deltacoronavirus. J. Virol..

[7] Van Regenmortel M.H.V., Fauquet C.M., Bishop D.H.L., Carstens E.B., Estes M.K., Lemon S.M., Maniloff J., Mayo M.A., McGeoch D.J., Pringle C.R. (2000). Virus Taxonomy: Classification and Nomenclature of Viruses Seventh Report of the International Committee on Taxonomy of Viruses.

[8] Malik Y.A. (2020). Properties of coronavirus and SARS-CoV-2. Malays. J. Pathol..

[9] Zumla A., Chan J.F., Azhar E.I., Hui D.S., Yuen K.Y. (2016). Coronaviruses-drug discovery and therapeutic options. Nat. Rev. Drug Discov..

[10] De Wit E., Van Doremalen N., Falzarano D., Munster V.J. (2016). SARS and MERS: Recent insights into emerging coronaviruses. Nat. Rev. Microbiol..

[11] World Health Organization Coronavirus Disease 2019 (COVID-19) Situation Report–89. https://www.who.int/emergencies/diseases/novel-coronavirus-2019/situation-reports/.

[12] Cui J., Li F., Shi Z. (2019). Origin and evolution of pathogenic coronaviruses. Nat. Rev. Microbiol..

[13] Alfaraj S.H., Al-Tawfiq J.A., Assiri A.Y., Alzahrani N.A., Alanazi A.A., Memish Z.A. (2019). Clinical predictors of mortality of middle east respiratory syndrome coronavirus (MERS-CoV) infection: A cohort study. Travel Med. Infect. Dis..

[14] Osipiuk J., Azizi S.A., Dvorkin S., Endres M., Jedrzejczak R., Jones K.A., Kang S., Kathayat R.S., Kim Y., Lisnyak V.G. (2021). Structure of papain-like protease from SARS-CoV-2 and its complexes with non-covalent inhibitors. Nat. Commun..

[15] Chen Y., Liu Q., Guo D. (2020). Emerging Coronaviruses: Genome structure, replication, and pathogenesis. J. Med. Virol..

[16] Hussain S., Pan J., Chen Y., Yang Y., Xu J., Peng Y., Wu Y., Li Z., Zhu Y., Tien P. (2005). Identification of novel subgenomic rnas and noncanonical transcription initiation signals of severe acute respiratory syndrome coronavirus. J. Virol..

[17] Czub M., Weingartl H., Czub S., He R., Cao J. (2005). Evaluation of modified vaccinia virus ankara based recombinant SARS vaccine in ferrets. Vaccine.

[18] Ramajayam R., Tan K., Liang P. (2011). Recent Development of 3C and 3CL protease inhibitors for anti-coronavirus and anti-picornavirus drug discovery. Biochem. Soc. Trans..

[19] Ren Z., Yan L., Zhang N., Guo Y., Yang C., Lou Z., Rao Z. (2013). The newly emerged SARS-like coronavirus Hcov-EMC also has an "Achilles’ Heel": Current effective inhibitor targeting a 3C-like protease. Protein Cell.

[20] van Boheemen S., de Graaf M., Lauber C., Bestebroer T.M., Raj V.S., Zaki A.M., Osterhaus A.D., Haagmans B.L., Gorbalenya A.E., Snijder E.J. (2012). Genomic characterization of a newly discovered coronavirus associated with acute respiratory distress syndrome in humans. mBio.

[21] De Diego M.L., Álvarez E., Almazán F., Rejas M.T., Lamirande E., Roberts A., Shieh W.J., Zaki S.R., Subbarao K., Enjuanes L. (2007). A severe acute respiratory syndrome coronavirus that lacks the E gene is attenuated *in Vitro* and *in Vivo*. J. Virol..

[22] Mortola E., Roy P. (2004). Efficient assembly and release of SARS coronavirus-like particles by a heterologous expression system. FEBS Lett..

[23] Kuo L., Masters P.S. (2003). The small envelope protein E is not essential for murine coronavirus replication. J. Virol..

[24] Ortego J., Ceriani J.E., Patiño C., Plana J., Enjuanes L. (2007). Absence of E protein arrests transmissible gastroenteritis coronavirus maturation in the secretory pathway. Virology.

[25] Ruch T.R., Machamer C.E. (2012). The coronavirus E protein: Assembly and beyond. Viruses.

[26] Siu Y., Teoh K., Lo J., Chan C., Kien F., Escriou N., Tsao S.W., Nicholls J.M., Altmeyer R., Peiris J.S.M. (2008). The M, E, and N structural proteins of the severe acute respiratory syndrome coronavirus are required for efficient assembly, trafficking, and release of virus-like particles. J. Virol..

[27] Rawlings N.D., Barrett A.J., Bateman A. (2010). MEROPS: The peptidase database. Nucleic Acids Res..

[28] Kuo C.J., Chi Y.H., Hsu J.T.A., Liang P.H. (2004). Characterization of SARS main protease and inhibitor assay using a fluorogenic substrate. Biochem. Biophys. Res. Commun..

[29] Fan K., Wei P., Feng Q., Chen S., Huang C., Ma L., Lai B., Pei J., Liu Y., Chen J. (2004). Biosynthesis, purification, and substrate specificity of severe acute respiratory syndrome coronavirus 3C-like proteinase. J. Biol. Chem..

[30] Hsu W.C., Chang H.C., Chou C.Y., Tsai P.J., Lin P.I., Chang G.G. (2005). Critical assessment of important regions in the subunit association and catalytic action of the severe acute respiratory syndrome coronavirus main protease. J. Biol. Chem..

[31] Tsai M.Y., Chang W.H., Liang J.Y., Lin L.L., Chang G.G., Chang H.P. (2010). Essential covalent linkage between the chymotrypsin-like domain and the extra domain of the SARS-CoV main protease. J. Biochem..

[32] Xia B., Kang X. (2011). Activation and maturation of SARS-CoV main protease. Protein Cell.

[33] Zhang L., Lin D., Sun X., Curth U., Drosten C., Sauerhering L., Becker S., Rox K., Hilgenfeld R. (2020). Crystal structure of SARS-CoV-2 main protease provides a basis for design of improved α-ketoamide inhibitors. Science.

[34] Jin Z., Du X., Xu Y., Deng Y., Liu M., Zhao Y., Zhang B., Li X., Zhang L., Peng C. (2020). Structure of Mpro from SARS-CoV-2 and discovery of its inhibitors. Nature.

[35] Roe M.K., Junod N.A., Young A.R., Beachboard D.C., Stobart C. (2021). Targeting novel structural and functional features of coronavirus protease nsp5 (3CLpro, Mpro) in the age of COVID-19. J. Gen. Virol..

[36] Kneller D.W., Phillips G., O’neill H.M., Jedrzejczak R., Stols L., Langan P., Joachimiak A., Coates L., Kovalevsky A. (2020). Structural plasticity of SARS-CoV-2 3CL Mpro active site cavity revealed by room temperature X-ray crystallography. Nat. Comm..

[37] Xue X., Yu H., Yang H., Xue F., Wu Z., Shen W., Li J., Zhou Z., Ding Y., Zhao Q. (2008). Structures of two coronavirus main proteases: Implications for substrate binding and antiviral drug design. J. Virol..

[38] Zhao Q., Li S., Xue F., Zou Y., Chen C., Bartlam M., Rao Z. (2008). Structure of the main protease from a global infectious human coronavirus, HCoV-HKU1. J. Virol..

[39] Anand K., Ziebuhr J., Wadhwani P., Mesters J.R., Hilgenfeld R. (2003). Coronavirus main proteinase (3CLpro) structure: Basis for design of anti-SARS drugs. Science.

[40] Su H., Zhou F., Huang Z., Ma X., Natarajan K., Zhang M., Huang Y., Su H. (2020). Molecular insights into small molecule drug discovery for SARS-CoV-2. Angew. Chem..

[41] Shi J., Wei Z., Song J. (2004). Dissection study on the severe acute respiratory syndrome 3C-like protease reveals the critical role of the extra domain in dimerization of the enzyme: Defining the extra domain as a new target for design of highly specific protease inhibitors. J. Biol. Chem..

[42] Anand K., Palm G.J., Mesters J.R., Siddell S.G., Ziebuhr J., Hilgenfeld R. (2002). Structure of coronavirus main proteinase reveals combination of a chymotrypsin fold with an extra alpha-helical domain. EMBO J..

[43] Muramatsu T., Takemoto C., Kim Y.T., Wang H., Nishii W., Terada T., Shirouzu M., Yokoyama S. (2016). SARS-CoV 3CL protease cleaves its C-terminal autoprocessing site by novel subsite cooperativity. Proc. Natl. Acad. Sci. USA.

[44] Chou C.Y., Chang H.C., Hsu W.C., Lin T.Z., Lin C.H., Chang G.G. (2004). Quaternary structure of the severe acute respiratory syndrome (SARS) coronavirus main protease. Biochemistry.

[45] Barrila J., Bacha U., Freire E. (2006). Long-range cooperative interactions modulate dimerization in SARS 3CLpro. Biochemistry.

[46] Chen S., Zhang J., Hu T., Chen K., Jiang H., Shen X. (2008). Residues on the dimer interface of SARS coronavirus 3C-like protease: Dimer stability characterization and enzyme catalytic activity analysis. J. Biochem..

[47] Chen S., Hu T., Zhang J., Chen J., Chen K., Ding J., Jiang H., Shen X. (2008). Mutation of Gly-11 on the dimer interface results in the complete crystallographic dimer dissociation of severe acute respiratory syndrome coronavirus 3C-like protease: Crystal structure with molecular dynamics simulations. J. Biol. Chem..

[48] Lin P.Y., Chou C.Y., Chang H.C., Hsu W.C., Chang G.G. (2008). Correlation between dissociation and catalysis of SARS-CoV main protease. Arch. Biochem. Biophys..

[49] Shi J., Sivaraman J., Song J. (2008). Mechanism for controlling the dimer-monomer switch and coupling dimerization to catalysis of the severe acute respiratory syndrome coronavirus 3C-like protease. J. Virol..

[50] Hu T., Zhang Y., Li L., Wang K., Chen S., Chen J., Ding J., Jiang H., Shen X. (2009). Two adjacent mutations on the dimer interface of SARS coronavirus 3C-like protease cause different conformational changes in crystal structure. Virology.

[51] Barrila J., Gabelli S.B., Bacha U., Amzel L.M., Freire E. (2010). Mutation of Asn28 disrupts the dimerization and enzymatic activity of SARS 3CLpro. Biochemistry.

[52] Yan F., Gao F. (2021). An overview of potential inhibitors targeting non-structural proteins 3 (PLpro and Mac1) and 5 (3CLpro/Mpro) of SARS-CoV-2. Comput. Struct. Biotechnol. J..

[53] Ziebuhr J., Gorbalenya A.E., Snijder E.J. (2000). Virus-encoded proteinases and proteolytic processing in the Nidovirales. J. Gen. Virol..

[54] Stobart C.C., Sexton N.R., Munjal H., Lu X., Molland K.L., Mesecar A.D., Denison M.R. (2013). Chimeric exchange of coronavirus NSP5 proteases (3CLpro) identifies common and divergent regulatory determinants of protease activity. J. Virol..

[55] Lu X., Lu Y., Denison M.R. (1996). Intracellular and in vitro-translated 27-kDa proteins contain the 3C-like proteinase activity of the coronavirus MHV-A59. Virology.

[56] Dai W., Zhang B., Jiang X.M., Su H., Li J., Zhao Y., Xie X., Jin Z., Peng J., Liu F. (2020). Structure-based design of antiviral drug candidates targeting the SARS-CoV-2 main protease. Science.

[57] Kanjanahaluethai A., Baker S.C. (2000). Identification of mouse hepatitis virus papain-like proteinase 2 activity. J. Virol..

[58] Donaldson E.F., Graham R.L., Sims A.C., Denison M.R., Baric R.S. (2007). Analysis of murine hepatitis virus strain A59 temperature-sensitive mutant TS-LA6 suggests that nsp10 plays a critical role in polyprotein processing. J. Virol..

[59] Hsu M.F., Kuo C.J., Chang K.T., Chang H.C., Chou C.C., Ko T.P., Shr H.L., Chang G.G., Wang A.H.J., Liang P.H. (2005). Mechanism of the maturation process of SARS-CoV 3CL protease. J. Biol. Chem..

[60] Tomar S., Johnston M.L., John S.E.S., Osswald H.L., Nyalapatla P.R., Paul L.N., Ghosh A.K., Denison M.R., Mesecar A.D. (2015). Ligand-Induced dimerization of middle east respiratory syndrome (MERS) coronavirus NSP5 protease (3CLpro) implications for NSP5 regulation and the development of antivirals. J. Biol. Chem..

[61] Li C., Qi Y., Teng X., Yang Z., Wei P., Zhang C., Tan L., Zhou L., Liu Y., Lai L. (2010). Maturation mechanism of severe acute respiratory syndrome (SARS) coronavirus 3C-like proteinase. J. Biol. Chem..

[62] Chen S., Jonas F., Shen C., Higenfeld R., Higenfeld R. (2010). Liberation of SARS-CoV main protease from the viral polyprotein: N-terminal autocleavage does not depend on the mature dimerization mode. Protein Cell.

[63] Krichel B., Falke S., Hilgenfeld R., Redecke L., Uetrecht C. (2020). Processing of the SARS-CoV pp1a/ab nsp7–10 region. Biochem. J..

[64] Deming D.J., Graham R.L., Denison M.R., Baric R.S. (2007). Processing of open reading frame 1A replicase proteins nsp7 to nsp10 in murine hepatitis virus strain A59 replication. J. Virol..

[65] Stobart C.C., Lee A.S., Lu X., Denison M.R. (2012). Temperature-sensitive mutants and revertants in the coronavirus nonstructural protein 5 protease (3CLpro) define residues involved in long-distance communication and regulation of protease activity. J. Virol..

[66] Stokes H.L., Baliji S., Hui C.G., Sawicki S.G., Baker S.C., Siddell S.G. (2010). A new cistron in the murine hepatitis virus replicase gene. J. Virol..

[67] Bosken Y.K., Cholko T., Lou Y.C., Wu K.P., Chang C.E.A. (2020). Insights into dynamics of inhibitor and ubiquitin-like protein binding in SARS-CoV-2 papain-like protease. Front. Mol. Biosci..

[68] Ibrahim T.M., Ismail M.I., Bauer M.R., Bekhit A.A., Boeckler F.M. (2020). Supporting SARS-CoV-2 papain-like protease drug discovery: In silico methods and benchmarking. Front. Chem..

[69] Fu Z., Huang B., Tang J., Liu S., Liu M., Ye Y., Liu Z., Xiong Y., Zhu W., Cao D. (2021). The complex structure of GRL0617 and SARS-CoV-2 PLpro reveals a hot spot for antiviral drug discovery. Nat. Commun..

[70] Ratia K., Saikatendu K.S., Santarsiero B.D., Barretto N., Baker S.C., Stevens R.C., Mesecar A.D. (2006). Severe acute respiratory syndrome coronavirus papain-like protease: Structure of a viral deubiquitinating enzyme. Proc. Natl. Acad. Sci. USA.

[71] Clasman J.R., Báez-Santos Y.M., Mettelman R.C., O’Brien A., Baker S.C., Mesecar A.D. (2017). X-ray structure and enzymatic activity profile of a core papain-like protease of MERS coronavirus with utility for structure-based drug design. Sci. Rep..

[72] Henderson J.A., Verma N., Harris R.C., Liu R., Shen J. (2020). Assessment of proton-coupled conformational dynamics of SARS and MERS coronaviruses papain-like proteases: Implication for designing broad-spectrum antiviral inhibitors. Chem. Phys..

[73] Klemm T., Ebert G., Calleja D.J., Allison C.C., Richardson L.W., Bernardini J.P., Lu B.G., Kuchel N.W., Grohmann C., Shibata Y. (2020). Mechanism and inhibition of the papain-like protease, PLpro, of SARS-CoV-2. EMBO J..

[74] Chou Y.W., Cheng S.C., Lai H.Y., Chou C.Y. (2012). Differential domain structure stability of the severe acute respiratory syndrome coronavirus papain-like protease. Arch. Biochem. Biophys..

[75] Barretto N., Jukneliene D., Ratia K., Chen Z., Mesecar A.D., Baker S.C. (2005). The papain-like protease of severe acute respiratory syndrome coronavirus has deubiquitinating activity. J. Virol..

[76] Ratia K., Pegan S., Takayama J., Sleeman K., Coughlin M., Baliji S., Chaudhuri R., Fu W., Prabhakar B.S., Johnson M.E. (2008). A noncovalent class of papain-like protease/deubiquitinase inhibitors blocks SARS virus replication. Proc. Natl. Acad. Sci. USA.

[77] Báez-Santos Y.M., Barraza S.J., Wilson M.W., Agius M.P., Mielech A.M., Davis N.M., Baker S.C., Larsen S.D., Mesecar A.D. (2014). X-ray structural and biological evaluation of a series of potent and highly selective inhibitors of human coronavirus papain-like proteases. J. Med. Chem..

[78] Báez-Santos Y.M., John S.E.S., Mesecar A.D. (2015). The SARS-coronavirus papain-like protease: Structure, function and inhibition by designed antiviral compounds. Antivir. Res..

[79] Hagemeijer M.C., Verheije M.H., Ulasli M., Shaltiel I.A., De Vries L.A., Reggiori F., Rottier P.J., De Haan C.A. (2010). Dynamics of coronavirus replication transcription complexes. J. Virol..

[80] Oostra M., Hagemeijer M.C., Van Gent M., Bekker C.P., Te Lintelo E.G., Rottier P.J., De Haan C.A. (2008). Topology and membrane anchoring of the coronavirus replication complex: Not all hydrophobic domains of nsp3 and nsp6 are membrane spanning. J. Virol..

[81] Kandeel M., Abdelrahman A.H., Oh-Hashi K., Ibrahim A., Venugopala K.N., Morsy M.A., Ibrahim M.A. (2020). Repurposing of FDA-approved antivirals, antibiotics, anthelmintics, antioxidants, and cell protectives against SARS-CoV-2 papain-like protease. J. Biomol. Struc. Dyn..

[82] Shin D., Mukherjee R., Grewe D., Bojkova D., Baek K., Bhattacharya A., Schulz L., Widera M., Mehdipour A.R., Tascher G. (2020). Papain-like protease regulates SARS-CoV-2 viral spread and innate immunity. Nature.

[83] Maiti B.K. (2020). Can papain-like protease inhibitors halt SARS-CoV-2 replication?. ACS Pharmacol. Transl. Sci..

[84] Lei J., Kusov Y., Hilgenfeld R. (2018). Nsp3 of coronaviruses: Structures and functions of a large multi-domain protein. Antivir. Res..

[85] Devaraj S.G., Wang N., Chen Z., Tseng M., Barretto N., Lin R., Peters C.J., Tseng C.T.K., Baker S.C., Li K. (2007). Regulation of IRF-3-dependent innate immunity by the papain-like protease domain of the severe acute respiratory syndrome coronavirus. J. Biol. Chem..

[86] Ratia K., Kilianski A., Báez-Santos Y.M., Baker S.C., Mesecar A. (2014). Structural Basis for the ubiquitin-linkage specificity and deisgylating activity of SARS-CoV papain-like protease. PLoS Pathog..

[87] Nicholson B., Leach C.A., Goldenberg S.J., Francis D.M., Kodrasov M.P., Tian X., Shanks J., Sterner D.E., Bernal A., Mattern M.R. (2008). Characterization of ubiquitin and ubiquitin-like-protein isopeptidase activities. Protein Sci..

[88] Dzimianski J.V., Scholte F.E., Bergeron É., Pegan S.D. (2019). ISG15: It’s complicated. J. Mol. Biol..

[89] Perng Y.C., Lenschow D.J. (2018). ISG15 in antiviral immunity and beyond. Nat. Rev. Microbiol..

[90] Sulea T., Lindner H.A., Purisima E.O., Menard R. (2005). Deubiquitination, a new function of the severe acute respiratory syndrome coronavirus papain-like protease?. J. Virol..

[91] Békés M., Ekkebus R., Ovaa H., Huang T.T., Lima C.D. (2016). Recognition of Lys48-linked di-ubiquitin and deubiquitinating activities of the SARS coronavirus papain-like protease. Mol. Cell.

[92] Clemente V., D’Arcy P., Bazzaro M. (2020). Deubiquitinating enzymes in coronaviruses and possible therapeutic opportunities for COVID-19. Int. J. Mol. Sci..

[93] McClain C.B., Vabret N. (2020). SARS-CoV-2: The many pros of targeting PLpro. Signal Transduct. Target Ther..

[94] Demain A.L. (2009). Antibiotics: Natural products essential for human health. Med. Res. Rev..

[95] Cani P.D., Knauf C. (2016). How gut microbes talk to organs: The role of endocrine and nervous routes. Mol. Met..

[96] Nicholson J.K., Holmes E., Kinross J., Burcelin R., Gibson G., Jia W., Pettersson S. (2012). Host-gut microbiota metabolic interactions. Science.

[97] O’Mahony S.M., Clarke G., Borre Y.E., Dinan T.G., Cryan J.F. (2015). Serotonin, tryptophan metabolism and the brain-gut-microbiome axis. Behav. Brain Res..

[98] Singh B.K., MacDonald C.A. (2010). Drug discovery from uncultivable microorganisms. Drug Discov. Today.

[99] Koehn F.E., Carter G.T. (2005). The evolving role of natural products in drug discovery. Nat. Rev. Drug Discov..

[100] Lavecchia A., Di Giovanni C. (2013). Virtual screening strategies in drug discovery: A critical review. Curr. Med. Chem..

[101] Tripathi A., Misra K. (2017). Molecular docking: A structure-based drug designing approach. JSM Chem..

[102] Wang Z., Jia J., Wang L., Li F., Wang Y., Jiang Y., Song X., Qin S., Zheng K., Ye J. (2020). Anti-HSV-1 activity of Aspergillipeptide D, a cyclic pentapeptide isolated from fungus *Aspergillus* sp. SCSIO 41501. Virol. J..

[103] Ma X., Zhu T., Gu Q., Xi R., Wang W., Li D. (2014). Structures and antiviral activities of butyrolactone derivatives isolated from *Aspergillus terreus* MXH-23. Ocean Univ. China.

[104] Ma X., Nong X., Ren Z., Wang J., Liang X., Wang L., Xi S. (2017). Antiviral peptides from marine gorgonian-derived fungus Aspergillus sp. SCSIO 41501. Tetrahedron Lett..

[105] Houda Sara N., Rachid B., Julio G., Jesus R.-S.M., Teresa V. (2020). In vitro antimicrobial, antiviral and cytotoxicity activities of *Aspergillus oryzae* isolated from El-Baida Marsh in Algeria. J. Drug Deliv. Ther..

[106] Koehler P., Bassetti M., Chakrabarti A., Chen S.C.A., Colombo A.L., Hoenigl M., Klimko N., Lass-Flörl C., Oladele R.O., Vinh D.C. (2021). Defining and managing COVID-19-associated pulmonary aspergillosis: The 2020 ECMM/ISHAM consensus criteria for research and clinical guidance. Lancet Infect. Dis..

[107] Zacharof M.P., Lovitt R.W. (2012). Bacteriocins produced by lactic acid bacteria a review article. APCBEE Procedia.

[108] Sayed A.M., Alhadrami H.A., El-Gendy A.O., Shamikh Y.I., Belbahri L., Hassan H.M., Abdelmohsen U.R., Rateb M.E. (2020). Microbial natural products as potential inhibitors of SARS-CoV-2 main protease (Mpro). Microorganisms.

[109] Rao P., Shukla A., Parmar P., Rawal R.M., Patel B., Saraf M., Goswami D. (2020). Reckoning a fungal metabolite, pyranonigrin A as a potential main protease (Mpro) inhibitor of novel SARS-CoV-2 virus identified using docking and molecular dynamic simulation. Biophys. Chem..

[110] Quimque M.T.J., Notarte K.I.R., Fernandez R.A.T., Mendoza M.A.O., Liman R.A.D., Lim J.A.K., Pilapil L.A.E., Ong J.K.H., Pastrana A.M., Khan A. (2021). Virtual screening-driven drug discovery of SARS-CoV2 enzyme inhibitors targeting viral attachment, replication, post-translational modification and host immunity evasion infection mechanisms. J. Biomol. Struct. Dyn..

[111] El-Hawary S.S., Mohammed R., Bahr H.S., Attia E.Z., El-Katatny M.H., Abelyan N., Al-Sanea M.M., Moawad A.S., Abdelmohsen U.R. (2021). Soybean-associated endophytic fungi as potential source for anti-COVID-19 metabolites supported by docking analysis. J. Appl. Microbiol..

[112] Prajapati J., Patel R., Goswami D., Saraf M., Rawal R.M. (2021). Sterenin M as a potential inhibitor of SARS-CoV-2 main protease identified from MeFSAT database using molecular docking, molecular dynamics simulation and binding free energy calculation. Comput. Biol. Med..

[113] Alam S., Sadiqi S., Sabir M., Nisa S., Ahmad S., Abbasi S.W. (2021). *Bacillus* species; a potential source of anti-SARS-CoV-2 main protease inhibitors. J. Biomol. Struct. Dyn..

[114] Naidoo D., Roy A., Kar P., Mutanda T., Anandraj A. (2021). Cyanobacterial metabolites as promising drug leads against the Mpro and PLpro of SARS-CoV-2: An in silico analysis. J. Biomol. Struct. Dyn..

[115] Balmeh N., Mahmoudi S., Fard N.A. (2021). Manipulated bio antimicrobial peptides from probiotic bacteria as proposed drugs for COVID-19 disease. Inform. Med. Unlocked..

[116] Rao P., Patel R., Shukla A., Parmar P., Rawal R.M., Saraf M., Goswami D. (2021). Identifying structural-functional analogue of GRL0617, the only well-established inhibitor for papain-like protease (PLpro) of SARS-CoV-2 from the pool of fungal metabolites using docking and molecular dynamics simulation. Mol. Divers..

[117] Bansal P., Kumar R., Singh J., Dhanda S. (2021). In silico molecular docking of SARS-CoV-2 surface proteins with microbial non-ribosomal peptides: Identification of potential drugs. J. Proteins Proteom..

[118] Gyebi G.A., Ogunro O.B., Adegunloye A.P., Ogunyemi O.M., Afolabi S.O. (2020). Potential inhibitors of coronavirus 3-chymotrypsin-like protease (3CLpro): An in silico screening of alkaloids and terpenoids from African medicinal plants. J. Biomol. Struct. Dyn..

[119] Sharma A., Goyal S., Yadav A.K., Kumar P., Gupta L. (2020). In-silico screening of plant-derived antivirals against main protease, 3CLpro and endoribonuclease, NSP15 proteins of SARS-CoV-2. J. Biomol. Struct. Dyn..

[120] Allam A.E., Amen Y., Ashour A., Assaf H.K., Hassan H.A., Abdel-Rahman I.M., Sayed A.M., Shimizu K. (2021). In silico study of natural compounds from sesame against COVID-19 by targeting Mpro, PLpro and RdRp. RSC Adv..

[121] Contreras-Puentes N., Alviz-Amador A. (2020). Virtual screening of natural metabolites and antiviral drugs with potential inhibitory activity against 3CL-PRO and PL-PRO. Biomed. Pharmacol..

[122] Van Santen J.A., Jacob G., Singh A.L., Aniebok V., Balunas M.J., Bunsko D., Neto F.C., Castaño-Espriu L., Chang C., Clark T.N. (2019). The natural products atlas: An open access knowledge base for microbial natural products discovery. ACS Central Sci..

[123] El-Neketi M., Ebrahim W., Lin W., Gedara S., Badria F., Saad H.E.A., Lai D., Proksch P. (2013). Alkaloids and polyketides from *Penicillium citrinum*, an endophyte isolated from the moroccan plant ceratonia siliqua. J. Nat. Prod..

[124] Riko R., Nakamura H., Shindo K. (2014). Studies on pyranonigrins-isolation of pyranonigrin E and biosynthetic studies on pyranonigrin A. J. Antibiot..

[125] Nong X.H., Wang Y.F., Zhang X.Y., Zhou M.P., Xu X.Y., Qi S.H. (2014). Territrem and butyrolactone derivatives from a marine-derived fungus *Aspergillus terreus*. Mar. Drugs.

[126] Wang B.T., Qi Q.Y., Ma K., Pei Y.F., Han J.J., Xu W., Li E.W., Liu H.W. (2014). Depside α -glucosidase inhibitors from a culture of the mushroom *Stereum hirsutum*. Planta Med..

[127] Elnagdy S., AlKhazindar M. (2020). The potential of antimicrobial peptides as an antiviral therapy against COVID-19. ACS Pharmacol. Transl. Sci..

[128] Maleki M.S.M., Rostamian M., Madanchi H. (2021). Antimicrobial peptides and other peptide-like therapeutics as promising candidates to combat SARS-CoV-2. ERATCK.

[129] Verma Y.K., Verma R., Tyagi N., Behl A., Kumar S., Gangenahalli G.U. (2021). COVID-19 and its therapeutics: Special emphasis on mesenchymal stem cells based therapy. Stem Cell Rev. Rep..

[130] Galmarini O.L., Stodola F.H., Raper K.B., Fennell D.I. (1962). Fonsecin, a naphthopyrone pigment from a mutant of *Aspergillus fonsecaeus*. Nature.

[131] Huang L.H., Xu M.Y., Li H.J., Li J.Q., Chen Y.X., Ma W.Z., Li Y.P., Xu J., Yang D.P., Lan W.J. (2017). Amino Acid-directed strategy for inducing the marine-derived fungus *Scedosporium apiospermum* F41-1 to maximize alkaloid diversity. Org. Lett..

[132] Peng J., Lin T., Wang W., Xin Z., Zhu T., Gu Q., Li D. (2013). Antiviral alkaloids produced by the mangrove-derived fungus *Cladosporium* sp. PJX-41. J. Nat. Prod..

[133] Adeoye A.O., Oso B.J., Olaoye I.F., Tijjani H., Adebayo A.I. (2020). Repurposing of chloroquine and some clinically approved antiviral drugs as effective therapeutics to prevent cellular entry and replication of coronavirus. J. Biomol. Struct. Dyn..

[134] Loll P.J., Upton E.C., Nahoum V., Economou N.J., Cocklin S. (2014). The high resolution structure of tyrocidine A reveals an amphipathic dimer. Biochim. Biophys. Acta..

[135] Fang A., Pierson D., Mishra S., Koenig D.W., Demain A.L. (1997). Gramicidin S production by *Bacillus brevis* in Simulated Microgravity. Curr. Microbiol..

[136] Lv Z., Chu Y., Wang Y. (2015). HIV protease inhibitors: A review of molecular selectivity and toxicity. HIV AIDS.

[137] Wang Z., Chen X., Lu Y., Chen F., Zhang W. (2020). Clinical characteristics and therapeutic procedure for four cases with 2019 novel coronavirus pneumonia receiving combined Chinese and Western medicine treatment. Biosci. Trends.

[138] Lim J., Jeon S., Shin H.Y., Kim M.J., Seong Y.M., Lee W.J., Choe K.W., Kang Y.M., Lee B., Park S.J. (2020). Case of the index patient who caused tertiary transmission of COVID-19 infection in Korea: The application of lopinavir/ritonavir for the treatment of COVID-19 infected pneumonia monitored by Quantitative RT-PCR. J. Korean Med. Sci..

[139] Tran L., Tam D.N.H., Elhadad H., Hien N.M., Huy N.T. (2021). Evaluation of COVID-19 protease and HIV inhibitors interactions. Acta Pharm..

[140] Raphael V.P., Shanmughan S.K. (2020). Computational evaluation of the inhibition efficacies of HIV antivirals on SARS-CoV-2 (COVID-19) protease and identification of 3D pharmacophore and hit compounds. Adv. Pharmacol. Sci..

[141] Bolcato G., Bissaro M., Pavan M., Sturlese M., Moro S. (2020). Targeting the coronavirus SARS-CoV-2: Computational insights into the mechanism of action of the protease inhibitors lopinavir, ritonavir and nelfinavir. Sci. Rep..

[142] Mahdi M., Mótyán J.A., Szojka Z.I., Golda M., Miczi M., Tozser J. (2020). Analysis of the efficacy of HIV protease inhibitors against SARS-CoV-2′s main protease. Virol. J..

[143] Razali R., Subbiah V.K., Budiman C. (2021). Technical data of heterologous expression and purification of SARS-CoV-2 proteases using Escherichia coli system. Data.

